# Two *hAT* transposon genes were transferred from Brassicaceae to broomrapes and are actively expressed in some recipients

**DOI:** 10.1038/srep30192

**Published:** 2016-07-25

**Authors:** Ting Sun, Susanne S. Renner, Yuxing Xu, Yan Qin, Jianqiang Wu, Guiling Sun

**Affiliations:** 1 Key Laboratory of Economic Plants and Biotechnology, Yunnan Key Laboratory for Wild Plant Resources, Kunming Institute of Botany, Chinese Academy of Sciences, Kunming 650201, China; 2 Institute of Plant Stress Biology, State Key Laboratory of Cotton Biology, Department of Biology, Henan University, Kaifeng 475004, China; 3 University of the Chinese Academy of Sciences, Beijing 100039, China; 4 Systematic Botany and Mycology, University of Munich (LMU), Munich 80638, Germany; 5 State Key Laboratory of Genetic Resources and Evolution, Kunming Institute of Zoology, Chinese Academy of Sciences, Kunming 650223, China

## Abstract

A growing body of evidence is pointing to an important role of horizontal gene transfer (HGT) in the evolution of higher plants. However, reports of HGTs of transposable elements (TEs) in plants are still scarce, and only one case is known of a class II transposon horizontally transferred between grasses. To investigate possible TE transfers in dicots, we performed transcriptome screening in the obligate root parasite *Phelipanche aegyptiaca* (Orobanchaceae), data-mining in the draft genome assemblies of four other Orobanchaceae, gene cloning, gene annotation in species with genomic information, and a molecular phylogenetic analysis. We discovered that the broomrape genera *Phelipanche* and *Orobanche* acquired two related nuclear genes (christened *BO* transposase genes), a new group of the *hAT* superfamily of class II transposons, from Asian Sisymbrieae or a closely related tribe of Brassicaceae, by HGT. The collinearity of the flanking genes, lack of a classic border structure, and low expression levels suggest that *BO* transposase genes cannot transpose in Brassicaceae, whereas they are highly expressed in *P. aegyptiaca*.

Horizontal gene transfer (HGT) refers to the transfer of genetic material to non-offspring genomes^1^,^2^,^3^. In prokaryotes, it is a vital source of evolutionary novelty. In eukaryotes, the role of HGT in physiological and ecological adaptations has become increasingly clear with examples now known from protists, fungi, animals, and plants^3^,^4^,^5^,^6^,^7^,^8^,^9^,^10^,^11^,^12^. For instance, a neochrome originating in hornworts was transferred horizontally to ferns, where it may have played a role in the diversification of modern ferns^5^. Most HGTs in plants involve mitochondrial DNA transferred to nuclear or mitochondrial genomes, while HGTs of nuclear and plastid DNA are rare, with the nuclear transfers mostly involving parasitic plants^6^,^7^,^8^,^9^,^10^,^11^,^12^. Thus, the root parasite *Striga hermonthica* (Orobanchaceae) has acquired an unknown protein-coding gene from its host *Sorghum* (Poaceae), apparently via an RNA intermediate^10^; the root parasite *Rafflesia cantleyi* (Rafflesiaceae) has acquired several dozen actively transcribed protein-coding genes from its obligate host *Tetrastigma rafflesiae* (Vitaceae)^6^; the root parasites *Orobanche* spp. and *Phelipanche* spp. (Orobanchaceae) and the shoot parasite *Cuscuta pentagona* (Convolvulaceae) have acquired *albumin* genes from legume hosts^11^, and *Phelipanche aegyptiaca* (Pers.) Pomel (formerly *Orobanche aegyptiaca* Pers.) and *Cuscuta australis* further acquired *strictosidine synthase*-like (*SSL*) genes from Brassicaceae hosts^12^. The frequency of HGT in parasitic plants is attributed to parasites establishing intimate connections with their hosts through haustoria that enable them to take up water, nutrients, and macromolecules including mRNAs^13^ and sometimes also DNA.

Transposable elements (TEs) constitute the main components of most eukaryotic genomes, occupying 3–20% of fungal, 3–45% of metazoan, 50% of human, and 80% or more of plant genomes^14^. They translocate to new positions in the same genome by either a copy-and-paste mechanism involving an RNA intermediate (class I TEs, retrotransposons) or by a cut-and-paste mechanism (class II TEs, transposons)^14^. Each class is subdivided into many ‘superfamilies’ based on shared structures, sequence homology, and transposition mechanisms^15^. Class I TEs usually cause genome expansion since they can duplicate repeatedly, and they are considered important sources of genome size differences among species^16^,^17^. Class II TEs exist in the majority of organisms and are the major form of transposable DNA in prokaryotes^18^. Their transposition is catalyzed by transposases that recognize short terminal inverted repeats (TIRs), excise the DNA transposon, and then ligate it into a new target site. In some cases, TEs may lose their transposition ability and are recruited as functional proteins by a process called TE domestication with or without gain of additional domains^18^.

Due to their mobility and capacity of transposition, transposable elements (TEs) are also involved in HGT. Horizontal transfers of both classes of TEs have been reported in fungi and animals, from which 199 transferred TEs were collected by the HTT-DB database with 1/3 involving class II transposons^19^. By searching 40 photosynthetic plant genomes, El Baidouri *et al*.^20^ recently detected 32 class I LTR retrotransposon transfer events in 26 species, with the transposons in some cases still functional. Some of these transfer events occurred between distantly related lineages, such as palms (Arecaceae) and grapevine (Vitaceae), tomato (Solanaceae) and bean (Fabaceae), or poplar (Salicaceae) and peach (Rosaceae)^20^. In contrast, only one horizontal transfer event of a class II transposon was identified in plants, involving two grass species^21^. Here we show that two novel nuclear-encoded transposon genes of class II were transferred from Brassicaceae into Orobanchaceae and are actively transcribed in both genera of Orobanchaceae, although there is little evidence that their homologs in Brassicaceae are transcribed.

## Results

### Two *hAT* transposon genes horizontally acquired by the common ancestor of *Orobanche* and *Phelipanche* from Brassicaceae

**Figure 1:**
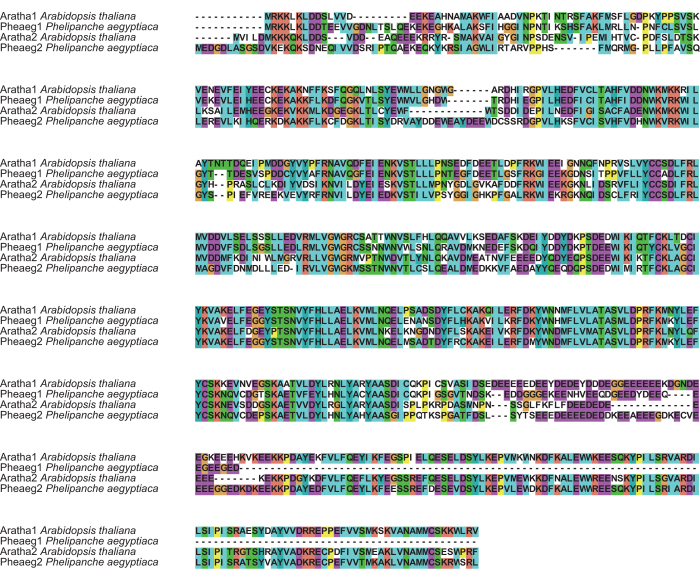
The protein alignment of the two foreign genes from *P. aegyptiaca* and the most similar proteins from *A. thaliana.* The species names of *Orobanche* and *Phelipanche* are indicated after the sequence IDs. Dashes indicate the sequences were incomplete or gaps introduced in the alignment. Background colors indicate the degree of conservation of the sites.

We developed a pipeline to perform transcriptome screening for foreign genes in *P. aegyptiaca*^12^. Using this screening pipeline, we found that two sequences in the *P. aegyptiaca* transcriptomes exhibited high similarities to the *A. thaliana hAT*-type DNA transposons: (i) one 1121-bp sequence (OrAeBC4_1751) showed 83% and 75% identities with AT3G17260 at the amino acid (AA) and nucleotide (NT) level, respectively, while being highly divergent from the most similar sequence in the Phrymaceae *Mimulus guttatus* (the highest identity was 28% at the AA level and there was no significant similarity at the NT level); (ii) another 1656-bp fragment (OrAeBC4_65399) had 85% and 65% identities with AT3G17290 at AA and NT level, while showing ~40% identity with the most similar homolog in *M. guttatus* at the AA level and no significant similarity at the NT level (Fig. 1). We then searched the partial genome sequences of *Orobanche austrohispanica, O. densiflora, O. gracilis*, and *O. rapum-genistae*^22^,^23^ and found that *O. austrohispanica* and *O. gracilis* also had these two genes; therefore, other species in *Orobanche* and *Phelipanche* probably have them as well.

**Figure 2:**
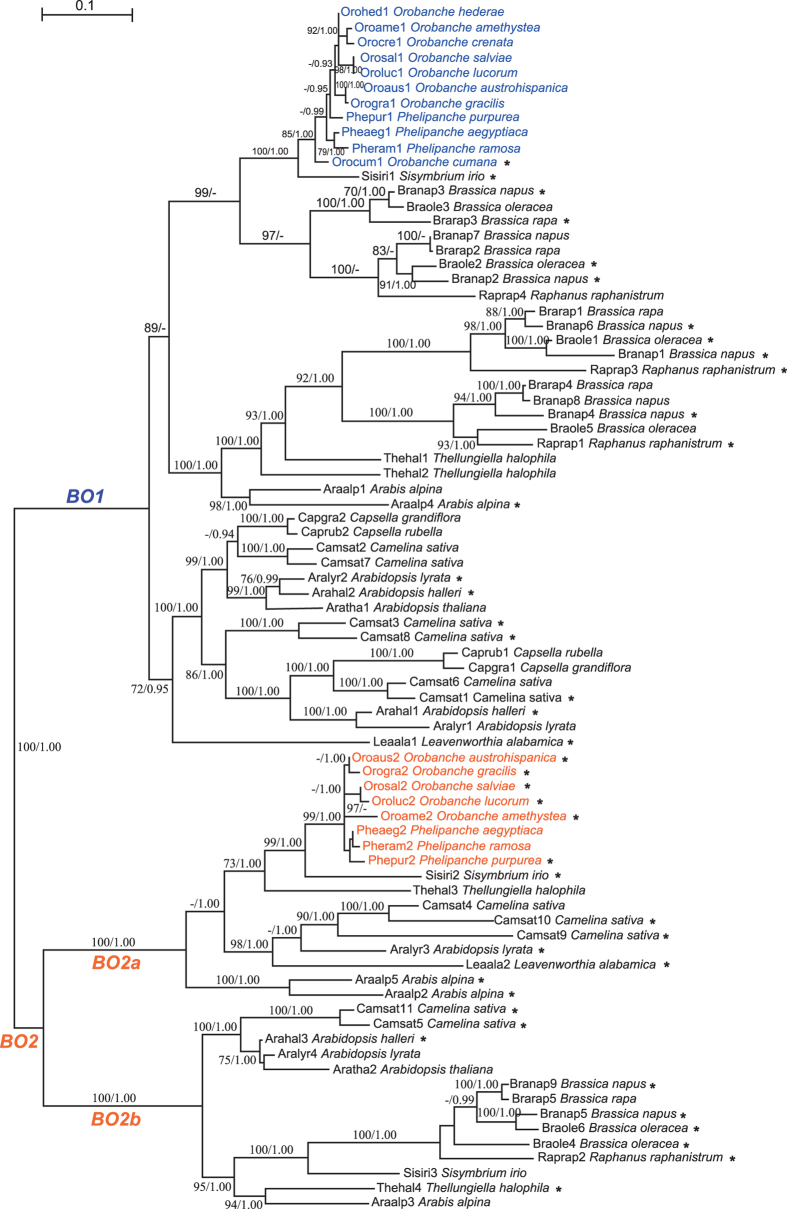
The protein tree of all the BOs in Brassicaceae and Orobanchaceae. Numbers at branches show bootstrap support values for maximum likelihood analysis (before the slash) and posterior probability values for MrBayes analysis (after the slash). Dashes indicate values lower than 70% in the maximum likelihood analysis and 0.90 in the MrBayes analysis. Asterisks after the species names indicate putative pseudogenes.

Genomic PCR amplification in seven *Orobanche* and three *Phelipanche* species (including *P. aegyptiaca*) using primers from the conserved regions resulted in amplification of both genes in these 10 species. We then obtained all sequences of Orobanchaceae and submitted to GenBank (Accession numbers: KM037755-6, KT892680-696, KU187277), and multiple sequence alignments showed that the two genes belong to two distinct types and that some gene copies have short indels, indicating that they have evolved into pseudogenes due to frameshift mutations in their coding regions (Fig. 2; Supplementary Fig. S1). All these data indicate that gene transfer events occurred between the common ancestor of *Orobanche*/*Phelipanche* and Brassicaceae. The genes were hereafter named *BO* (Brassicaceae and Orobanchaceae) to reflect their patchy distribution.

To gain insight into the direction of the horizontal transfer and its evolutionary time, we investigated the distribution of the *BO* transposase genes in the genomes of 18 sequenced species (Supplementary Table S1) that span the core Brassicaceae^24^,^25^ (320 genera, 3660 species, 49 tribes) as well as the available genomes of other parasitic flowering plants. In most Brassicaceae genome sequences, the *BO* genes have not been well annotated. We therefore extended the core regions of the *BO* gene candidates in Brassicaceae species to 5 kb in both directions and annotated the *BO* genes manually, using different gene prediction software. ORF Finder and GENSCAN successfully predicted the coding regions of most of the genes. To detect pseudogenized *BO* genes, we used three additional programs, Augustus, GeneSeqer, and Transeq in the EMBOSS package, and combined multiple sequence alignment with other well-annotated genes for optimal prediction. Except for three species, all analyzed Brassicaceae possess 2 to 11 *BO* genes (Supplementary Table S1), with half of them apparently pseudogenized, as determined by premature stop codons or indels causing frame shifts (Fig. 2). That we failed to detect the *BO* genes in three of the species may be due to the incomplete genome sequence data. The same procedure was used to search the genomes of non-Brassicaceae species in the Phytozome database, but no *BO* genes were found.

To detect *BO* genes in other parasitic plants, we used the *BO* genes from *A. thaliana* and *P. aegyptiaca* as queries to BLAST-search relevant transcriptome assemblies, including the assemblies of the Convolvulaceae *Cuscuta australis* and *C. pentagona*, the assemblies of the Orobanchaceae *Striga hermonthica* (StHeBC2), *Triphysaria versicolor* (TrPuRnBC1) and *Triphysaria pusilla* (TrVeBC2) in PPGP, the Lauraceae *Cassytha filiformis*, and the Apodanthaceae *Pilostyles thurberi* in the 1KP project (http://onekp.com/project.html). This yielded only sequences with low identities, and subsequent phylogenetic analysis indicated that none of them clustered with the *BO* genes.

A phylogeny of the protein sequences encoded by the *BO* transposase genes and proteins from other angiosperms whose genomes have been sequenced is shown in Supplementary Fig. S2. The BO proteins and their relatives form two clusters. Cluster I includes the BOs from Brassicaceae and Orobanchaceae and several sequences from peach (Prunus, Rosaceae). Cluster II contains the remaining angiosperms, the *A. thaliana DAYSLEEPER* gene^26^,^27^, and other *DAYSLEEPER*-like genes in three statistically well-supported groups (Supplementary Fig. S2). Within Cluster I, there are two groups, each containing at least one gene from the Brassicaceae and Orobanchaceae species. A highest posterior probability tree and a maximum likelihood tree obtained from all *BO* genes of 11 Orobanchaceae and 14 Brassicaceae (the *BO* genes in *Thellungiella parvula* were excluded because of their 100% identity with the homologs from *T. halophila*) both show distinct *BO1* and *BO2* clades (Fig. 2). The *BO1* genes in Brassicaceae have more copies than do the *BO2* genes, and the *BO2* genes further form two clusters, *BO2a* and *BO2b*, each containing genes from most Brassicaceae species, suggesting ancient divergence. Notably, each *BO* gene clustered with homologs from *Sisymbrium irio* (Fig. 2). This suggests that the two *BO* genes may have been laterally transferred into the common ancestor of the *Orobanche*/*Phelipanche* clade from *Sisymbrium*, a small Old World genus that is the sole member of the tribe Sisymbrieae^24^, or from a closely related species.

### The *BO* transposase genes constitute a new member of the *hAT* transposon superfamily

**Figure 3:**
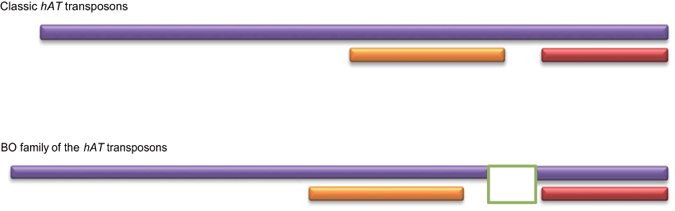
Domain structure comparison of a classic *hAT* family transposon with the *BO* transposase gene family in Brassicaceae, *Orobanche*, and *Phelipanche.* The ribonuclease H-like domain, the *hAT*-like transposase domain and the *hAT* dimerization domain are in purple, orange and red, respectively. The insertion position in the ribonuclease H-like domain of the BOs is displayed as a rectangle.

To gain further insight into the function and evolution of the *BO* transposase genes, we searched for their domains using InterProScan. Three domains were found: a *hAT* dimerization, a *hAT*-like transposase, and a ribonuclease H-like domain. The *hAT* dimerization domain is ~80 AA long and located in the C-terminus; the *hAT*-like transposase domain has ~100 AA and is located in the medial region; the ribonuclease H-like domain is ~500 AA long and covers the above two domains (Fig. 3; Supplementary Fig. S3). The conserved *hAT* dimerization domain is a hallmark of the *hAT* transposons and has been shown to be involved in dimerization as well as additional interaction functions^28^. The ribonuclease H-like domain is constituted of a three-layer alpha/beta/alpha fold and exists in some transposases, ribonucleases, exonucleases, and retroviral integrases^29^. Notably, the ribonuclease H-like domain in the BOs contains an insertion of 12-71 AA that lies between the *hAT*-like transposase domain and the *hAT* dimerization domain (Fig. 3; Supplementary Fig. S3). These results indicate that the *BO*s belong to a new class of the *hAT* superfamily.

Given that the BOs are closely related to the *A. thaliana* DAYSLEEPER protein (Supplementary Fig. S2), which contains an additional C2H2 type BED-zinc finger domain in the N terminus ahead of the three classic domains, we searched for this domain in other sequences of cluster II and found that most proteins possess this additional domain. Knip *et al*.^30^ also identified these genes as *DAYSLEEPER*-like or *SLEEPER.* The *hAT* family members have been categorized as *Ac*, *Buster*, and *BuT2*, and the DAYSLEEPER protein together with the Ac and TAM3 belongs to the Ac group. To investigate how the BOs are related to these Ac group members, we first used the two BOs in *A. thaliana* as representatives to compare with the typical Ac of maize and TAM3 of snapdragon. They showed 33% and 37% sequence identities with Ac of maize (GenBank accession number ACG45782) and 32% and 20% sequence identities with TAM3 of snapdragon (GenBank accession number CAA38906) at AA level. Sequence alignment also showed that the BOs are less similar to Ac and TAM3 than to DAYSLEEPER-like proteins. This supports that the *BO*s in *Orobanche*, *Phelipanche*, and Brassicaceae are a new *hAT* superfamily member, closely related to the *DAYSLEEPER*-like genes.

### *BO* genes in *P. aegyptiaca* and *O. cumana* are actively transcribed

**Figure 4:**
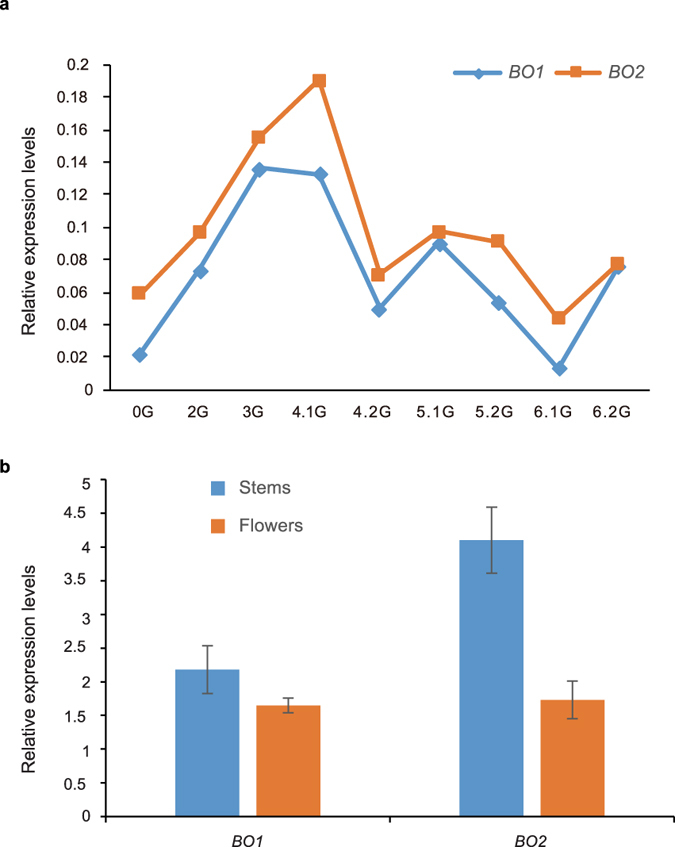
Expression levels of the two *BO* transposase genes in *P. aegyptiaca*. **(a)** Expression levels at different developmental stages (PPGP) estimated from RNA-seq data with ACTIN as the reference gene. **(b)** Expression levels (±SE, n = 3) of the two *BO* genes in stems and flowers assessed by qRT-PCR.

The presence of the two *BO* genes in the RNA-seq data of *P. aegyptiaca* indicates that they are actively transcribed. To determine how the expression level changes in different tissues at different developmental stages, we mapped all clean paired-end reads to our own Trinity assembly of the *P. aegyptiaca* transcriptome. The FPKM values of the *BO1* gene ranged from 2.1 to17.4 and those of the *BO2* genes were from 6.0 to 26.5. Figure 4a shows the relative expression levels of *BO1* and *BO2* with *actin* as the reference gene. Except in flowers, the *BO2* gene had higher expression levels than the *BO1* gene in all tissues^31^ (0G, seeds imbibed, pre-germination; 2G, seedling after exposure to haustorial induction factors; 3G, haustoria attached to host root, early penetration stages, pre-vascular connection; 4.1G, early established parasite, parasite vegetative growth after vascular connection; 4.2G, spider stage; 5.1G, pre-emerged leaves and stems; 5.2G, pre-emerged roots; 6.1G, post emergence from soil - vegetative structures, leaves/stems; 6.2G, post emergence from soil - reproductive structures, floral buds), and a comparison among different development stages showed that both *BO1* and *BO2* exhibited greater expression levels in the 3G and 4.1G stages than in the other stages.

We further assessed the transcription levels of the *BO* transposase genes in flowers and stems of *P. aegyptiaca* by quantitative real time-PCR (qRT-PCR), which provides accurate assessments of gene expression. In flowers and stems, the expression levels of the two *BO* genes were similar to those of the reference gene *actin* (Ct_*actin*_ was 24.2 ± 0.09 and 25.2 ± 0.32, Ct_*BO1*_ was 23.6 ± 0.19 and 24.3 ± 0.04, and Ct_*BO2*_ was 25.0 ± 0.37 and 24.5 ± 0.28 in flowers and stems, respectively). The *BO1* gene presented similar expression levels in stems and flowers, while the expression level of the *BO2* gene in stems was more than 1-fold higher than in flowers (Fig. 4b). To determine whether the *BO* transposase genes are expressed in *O. cumana*, we performed RT-PCR using the stem and flower tissues with the same primer pair as used in qRT-PCR. It produced a band with the expected size, and the identity of which was confirmed by sequencing.

These transcriptional data indicate that both *BO* genes have relatively high transcript levels in *Phelipanche* and *Orobanche* and that different pathways regulate their transcription.

### The *BO* genes in Brassicaceae show low transcriptional activity

The transcriptional levels of the *BO* genes in Brassicaceae were investigated by searching the microarray dataset of *A. thaliana* in Genevestigator and the NCBI EST and SRA databases. The Genevestigator analysis indicated that the *BO*s in *A. thaliana* are transcribed at low levels in most tissues except that the *BO1* gene (AT3G17260) is expressed at medium level in sperm cells (Supplementary Fig. S4). No ESTs corresponding to the two *BO* genes of *A. thaliana* were found in the Phytozome database. We further searched multiple RNA-seq datasets of *A. thaliana* (ecotype Col-0) in the SRA database but found no transcription evidence of the two *BO* genes, except a *BO2* RPKM value of 0.48 in one dataset^32^. Data-mining in the Phytozome revealed that two *BO1* genes in *Capsella rubella* (Caprub1 and Caprub2), one *BO1* gene (Thehal1) and two *BO2* genes in *Thellungiella halophila* (Thehal3 and Thehal4), and two *BO1* genes in *Brassica rapa* (Brarap1 and Brarap2) have very low expression levels. No expression data were available for *S. irio*, and searching the SRA database indicated that except for two *BO1* genes in *Brassica napus* (Branap6 and Branap7) and all four *BO* genes in *Raphanus raphanistrum* (all these genes’ RPKM values were <0.5), other genes had no mapped reads in Brassicaceae. The low transcriptional status, together with pseudogenization in half of the *BO* genes, suggest that the *BO* genes in Brassicaceae are generally not actively transcribed and unlikely to be able to transpose.

### The *BO* transposase genes and their flanking regions show synteny in Brassicaceae species

**Figure 5:**
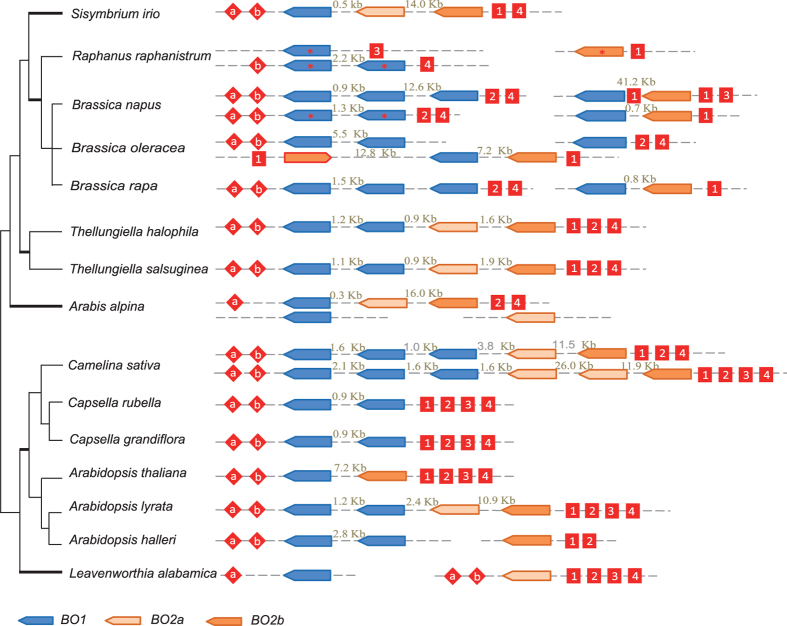
The chromosomal location and synteny of Brassicaceae *genes* encoding the BO family of *hAT* transposons. Intergenic regions are indicated above the dash lines. Red rhombuses on the upstream and red rectangles on the downstream flanking regions of the *BO* genes with letters or numbers indicate the following conserved genes: a, AT3G17205; b, AT3G17240; 1, AT3G17300; 2, AT3G17310; 3, AT3G17320; 4, AT3G17340.

We also investigated the chromosomal location of the *BO* transposase genes and the synteny of their flanking regions in the 15 available Brassicaceae genomes. Most *BO* genes were arranged in tandem or very close to each other (Fig. 5). Detailed inspection indicated that the ORF directions are the same for all but one *BO2* gene in *B. oleracea* (Fig. 5). As for the synteny of genes flanking the *BO* genes, we used six conserved genes with transcript activity in the flanking regions of *BO1-BO2* in *A. thaliana* to search against the other 14 Brassicaceae genomes. Of the six genes, two are located in the upstream flanking region of the *BO1*-*BO2* structure; the other four genes are located in the downstream flanking region of the *BO1*-*BO2* structure. This indicates that most flanking genes retained synteny around the *BO* genes except for a few losses (Fig. 5), such as the two-gene loss in the downstream flanking region of the *BO* gene in *S. irio*.

To further investigate whether the *BO* genes in Orobanchaceae and Brassicaceae can transpose, we inspected their flanking regions. Active *hAT* genes have short flanking regions that are converted to the donor sequence upon insertion (target site duplication, TSD) and short terminal inverted repeats (TIRs) (TSD-TIR-transposase-TIR-TSD). The length of the TSD structure is reported to be 8 bp for the *hAT* superfamily genes^33^. We failed to obtain the flanking regions of the *BO* genes in Orobanchaceae and therefore focused on the Brassicaceae *BO* genes. Transpolator was used to identify the 8-bp TSD and TIR structures in the flanking regions of the Brassicaceae *BO* genes. Different TSD-TIR structures were found but few had 8 bp-long TSDs, nor could their consensus sequence be obtained, including the “(T/C)A(A/G)NG” consensus sequence proposed by Rubin *et al*.^33^. The collinearity of the flanking genes and the absence of the typical TSD-TIR structures suggest that the *BO* genes in Brassicaceae did not have a history of transposition.

## Discussion

The *hAT* superfamily (named after the families: hobo from *Drosophila melanogaster*, Activator (Ac) from maize, and Tam3 from snapdragon) of class II TEs is widely distributed in fungi, animals, and plants^34^, and phylogenetic analyses suggest that it is ancient, predating the early stages of the divergence of these kingdoms^33^. In animals, *hAT* has been horizontally transferred among species of *Drosophila*^35^,^36^,^37^, and a novel *hAT* transposon having the 8-bp TSDs with the characteristic of recent transposition has been found in the silkworm, *Bombyx mori*, and in a Triatominae vector of the Chagas parasite, *Rhodnius prolixus*^38^. Prior to this study, no transfer events involving the transposase genes of the *hAT* superfamily had been reported in plants. By HGT screening in *P. aegyptiaca* transcriptomic data, we discovered two *BO* genes with high similarities to their homologs in Brassicaceae. Because parasitic plants can take up mRNAs from their hosts^39^, foreign mRNAs in *P. aegyptiaca* might be a contamination from Brassicaceae hosts, since *P. aegyptiaca* sometimes parasitizes *Arabidopsis*. To exclude this possibility, we carried out BLAST searches against the draft genomes of four *Orobanche* species and did genomic PCRs in seven *Orobanche* and three *Phelipanche* species, and this revealed two *BO* genes in the 11 species. Subsequent multiple sequence alignments clearly showed that the *BO* genes form two clusters, with each present in all tested *Orobanche* and *Phelipanche* (Supplementary Fig. S1).

By gene annotation and homology search, we found that the *BO* genes are absent from bacteria, viruses, and archaea, and are unique to Brassicaceae and the Orobanchaceae genera *Orobanche* and *Phelipanche*. Based on phylogenetic analysis of species representing the core Brassicaceae^25^,^40^, it appears that the two *BO* genes in *Orobanche* and *Phelipanche* are likely to be derived from *Sisymbrium*, a small Old World genus that is the sole member of Sisymbrieae^24^,^25^, or certain closely related species. Future genome sequences of species from the tribes Thelypodieae (26 genera, 224 species) and Isatideae (5 genera, 90 species), which are the closest relatives of Sisymbrieae, will further clarify the origin of the *BO* genes in Orobanchaceae. It is unlikely that the *BO* genes were instead transferred from Orobanchaceae to Brassicaceae, given that the *BO* transcripts were not found in *Striga hermonthica* (Orobanchaceae) and that the *BO*s spread from Sisymbrieae (or a close relative) to all the other core Brassicaceae species is implausible.

**Figure 6:**
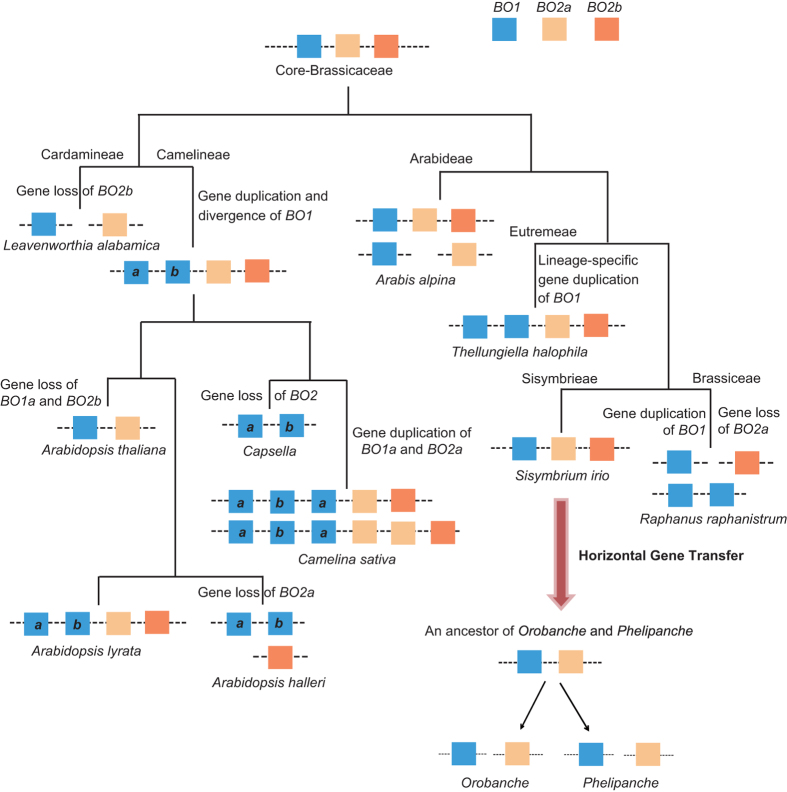
Scenario for the evolutionary history of the *BO* transposase genes in Brassicaceae and their transfer from an *S. irio*-like plant to *Orobanche* and *Phelipanche.* The genes *BO1, BO2a* and *BO2b* are indicated as blocks in different colors. The *a* and *b* in tribe Camelineae indicate the gene duplication and subsequent divergence of the *BO1* gene. The lineage specific gene duplication of the *BO1* gene in the tribe Eutremeae and the tribes Brassiceae and Sisymbrieae is shown in the same color. The two small unsampled tribes closest to Sisymbrieae are discussed in the text.

A scenario explaining the two foreign *BO* genes in *Orobanche* and *Phelipanche* is given in Fig. 6. It assumes a single gene transfer event involving the *BO1-BO2* DNA fragment from an ancestor, such as *S. irio*, to the common ancestor of minimally *Orobanche* and *Phelipanche* (our sampling of eight *Orobanche* and three *Phelipanche* is insufficient to exclude an earlier uptake). Orobanchaceae are a mostly temperate zone family of ca. 2000 species in 89 to 99 genera, and the earliest lineages of Orobanchaceae are not parasitic^41^. Attempts to confirm whether the *BO1* and *BO2* genes in Orobanchaceae are arranged in tandem failed, suggesting either long intervals between the *BO1* and *BO2* genes or that they are located on different chromosomes. Given the possibility of genome structure rearrangement in the recipients, different chromosomal locations or a distant placement on the same chromosome do not rule out an initial simultaneous acquisition.

The gene arrangement *BO1-BO2a-BO2b* likely was present in the ancestor of core Brassicaceae, judging from the analyzed genomes, which represent eight of their 49 tribes of the family and span the root of core Brassicaceae^25^,^40^. The *BO2b* gene was lost in the Cardamineae; gene duplication and divergence of *BO1* occurred in the Camelineae; and gene loss in *A. thaliana, A. halleri*, and *Capsella*, with further gene duplication in *C. sativa*; lineage-specific gene duplication of the *BO1* gene also occurred before the split of *Raphanus* and *Brassica*.

The HGT event of *BO* genes from Brassicaceae to *Orobanche* and *Phelipanche* must have occurred before the divergence of *Orobanche* and *Phelipanche.* Based on molecular-clock dating, *Orobanche* and *Phelipanche* diverged from each other 38 million years ago (Ma), with a 95% confidence interval of 16–45 Ma^23^. In our *BO* gene phylogeny, Orobanchaceae genes cluster with *Sisymbrium* with a stem age of 18 to 25 million years^40^ (Fig. 2; Supplementary Fig. S2). The related tribes Thelypodieae and Isatideae, from whose species genome information is not yet available and who could also have been the donor of the *BO* genes, shared a common ancestor with Sisymbrieae around 32 to 33 Ma^40^. The HGT might thus have happened between 16 and 33 Ma.

Parasite/host plant systems, which all involve distantly related species, are prone to HGT, because of the tight physical connections in the haustoria, which in the case of endoparasites, such as *Rafflesia* and *Pilostyles*, involve a network of parenchyma cells living inside the host^42^,^43^. DNA fragments are therefore transported between parasites and hosts and occasionally become integrated into parasite genomes (*Introduction*), with end-joining, homologous recombination, and virus-mediated integration as possible mechanisms^1^,^3^,^4^. In line with this, the horizontal transfer of TEs has been documented^20^,^21^, but so far TE-mediated integration of laterally transferred genes has not be found. Such transfer probably is facilitated by the transposons’ ability to incorporate into a recipient genome. This could have involved translation in the recipient cells of transferred *BO* genes contained in a DNA fragment. In plants, DNA transferred to stem tissue can be passed to the next generation because floral meristems can develop in leaf axils.

The *BO* genes in Brassicaceae likely lost their transposition ability just after their origin as evidenced by: 1) their flanking regions still being syntenic in almost all the investigated Brassicaceae species, indicating that they did not transpose at all; 2) their lack of the classic TSD border structure required for the recognition of transposases; 3) their closest relatives being the SLEEPER genes (Fig. S2), which are domesticated *hAT* transposons that are involved in the normal plant growth of both *A. thaliana*^26^,^27^ and rice^30^; 4) both *BO* genes in *A. thaliana* being considered domesticated^44^; and lastly by 5) many brassicaceous *BO* genes being pseudogenes (Fig. 2) and all *BO*s having very little transcriptional activity. Thus, the transfer of these *BO* genes from Brassicaceae to Orobanchaceae mostly likely did not involve any TE transposition, but instead another mechanism. The relatively high transcript levels of the *BO* genes in Orobanchaceae could either result from a location in the Orobanchaceae genomes, where transcription is especially active, or from them having gained new functions.

## Methods

### Data sources

All transcriptome assemblies of species of *Phelipanche* (the sole available *Phelipanche* species in the Parasitic Plant Genome Project (PPGP) is *P. aegyptiaca*, erroneously called *Orobanche aegyptiaca*), *Striga*, and *Triphysaria*, and the pair-end RNA-seq data from *P. aegyptiaca* were retrieved from the PPGP website (http://ppgp.huck.psu.edu). The protein database used by AlienG^45^ for BLAST searches was the NCBI non-redundant (nr) database (Sep., 2012) and included all predicted proteins from 22 plant genomes available in the Phytozome database version 9.0^46^. The nucleotide database used was the NCBI nucleotide (nt) collection with all predicted transcripts of 22 plant genomes from Phytozome. The databases in the 1 KP project (http://www.onekp.com/project.html), including the transcriptome assemblies from *Cuscuta pentagona* Englm., *Cassytha filiformis* L., and *Pilostyles thurberi* A. Gray), PlantGDB (http://www.plantgdb.org), and SOL Genomics Network (http://solgenomics.net), were searched online. Our own RNAseq assembly of *Cuscuta australis* R.Br.^12^ was searched locally. The genomic sources of the 18 Brassicaceae species are provided in Supplementary Table S1.

### Transcriptome screening for foreign genes in *Phelipanche aegyptiaca*

Transcriptome screening for the transferred genes was performed according to the procedure described previously^12^. Briefly, the contamination from the host transcripts was first excluded by only choosing the genes expressed in seedlings not yet attached to the host; second, the coding regions were predicted, and only sequences with a contiguous amino acid length >100 AA were kept for AlienG^45^ analysis; finally, after filtering out those with highest similarities to the genes in *Mimulus guttatus* at the nucleotide acid level, the candidates predicted by AlienG with score ratios of the first non-Lamiales hit to the first Lamiales hit >1.2 were retained for manual identification of horizontally transferred genes.

### Identification of the foreign *hAT* transposon in Orobanchaceae and the closest homologs in Brassicaceae and other plants

The foreign genes detected in *P. aegyptiaca* by searching the PPGP data were two partial gene fragments. To obtain longer sequences, we downloaded the paired-end data from PPGP website and reassembled them using the Trinity software^47^ following the standard procedure. We also searched (BLASTn and tBLASTn) the partial draft genomes from other species of *Orobanche*, *O. densiflora* Salz. ex Reut*. O. austrohispanica* M.J.Y.Foley*, O. rapum-genistae* Thuill., and *O. gracilis* Sm.^23^. Hits with identity values >30% were extracted and distantly divergent sequences in the phylogeny tree were removed from further analysis. The closest homologs in the genomes of 15 Brassicaceae species were originally found by obtaining the sequence segments using the two *BO* transposase genes of *A. thaliana* (AT3G17260 and AT3G17290) as queries, which were extended in both directions to 5 kb for analysis in the next step.

Four types of software were used to identify the genes, including ORF Finder (NCBI)^48^, GENSCAN^49^ (http://genes.mit.edu/GENSCAN.html), Augustus^50^ (http://bioinf.uni-greifswald.de/augustus/submission), Transeq (http://www.ebi.ac.uk/Tools/st/emboss_transeq/), and GeneSeqer^51^ (http://www.plantgdb.org/cgi-bin/GeneSeqer/index.cgi), with *Arabidopsis* amino acid sequences as references when necessary. The pseudogenes were determined by the premature stop codons or indels causing coding frame shifts. The same procedure was used to identify the closest homologs with the two *A. thaliana BO* transposase genes in other genomes of relevant species.

### Phylogenetic analyses of the new *hAT* transposases

The protein homologs are extracted from representative species genomes in the Phytozome database and our own assembly of *P. aegyptiaca* by BLAST searching using the two *BO* transposase genes of *A. thaliana* as queries with the score value threshold set to 40. The sequences were aligned with ClustalX v2.1^52^, visually inspected, and manually refined. Gaps and ambiguously sites were removed from the alignment. ModelGenerator (v_851) was used to find the best-fitting model of protein substitution^53^. Phylogenetic trees were inferred under maximum likelihood optimization using PHYML v3.0^54^ and under Bayesian optimization using MrBayes v3.2.5^55^. Markov chain Monte Carlo chains were run with the default four chains and using two million generations, sampling trees every 200 generations. After discarding the first 5,000 trees (that is, half the trees) as burn-in, a consensus tree with the posterior probability support values for all clades was calculated from the remaining trees. Trees were viewed and edited using NJplot^56^.

### Expression level estimation of *hAT*-like genes in *Phelipanche aegyptiaca*, *Arabidopsis thaliana*, and other Brassicaceae

The expression levels of the two foreign *hAT* transposons in *P. aegyptiaca*, *BO1* and *BO2*, in different tissues and at different developmental stages were estimated using RSEM^57^ with *actin* as the reference gene. We mapped all clean reads to the Trinity assembly, and to obtain normalized expression levels we used the fragments per kilo base of exon per million fragments mapped (FPKM) values of the sequences. The *actin* gene was found with nine transcript isoforms, of which two contained the primer sequences Actin-F/Actin-R (below) and the sum of their FPKM values was used as the reference. Web-based expression analysis of the two *BO* transposase genes in *A. thaliana* (AT3G17260 and AT3G17290) in different tissues, different development stages, and under various treatments was performed using GENEVESTIGATOR v3 (https://www.genevestigator.com/gv/)^58^. Estimation of RNAseq-based transcriptional levels was also performed in other Brassicaceae by blast searches against randomly selected SRA datasets.

### Genomic PCR amplification of the two *BO* transposase genes in seven *Orobanche* and three *Phelipanche* species

Seeds of *P. aegyptiaca* and *Orobanche cumana* Wallr. were inoculated near the roots of 50 days-old tobacco for establishing parasite infection. Two-month-old *P. aegyptiaca* and *O. cumana* were sampled and stored at −80 °C. Samples of the following Orobanchaceae, *O. amethystea* Thuill., *O. crenata* Forssk., *O. salvia* F.W.Schultz, *O. hederae* Duby, *O. lucorum* A.Braun ex F.W.Schultz, *O. gracilis* Sm., *Phelipanche purpurea* (Jacq.) Soják, and *P. ramosa* (L.) Pomel were collected in the Munich Botanical Garden. Total DNA was extracted using a modified cetyltrimethylammonium bromide (CTAB) method. For genomic PCR, all *BO1* genes were obtained with OhAT-AF2/OhAT-AR primer pair, while the *BO2* genes were amplified by using multiple primer pairs depending on species (Supplementary Table S2). Two primer pairs B2F/B2R and C3F/C3R, were used to get two overlapping regions of the *BO2* in *O. cumana*. PCR primer sequences and the GenBank accession numbers of the *BO* genes in the 10 Orobanchaceae species with the herbarium voucher information are indicated in Supplementary Table S2. These primers were designed according to the multiple sequence alignment of the *BO* genes extracted from the transcriptome and draft genomic data above. Amplification products with expected sizes were sequenced directly or after cloning.

### qRT-PCR of the *BO* genes in *Phelipanche aegyptiaca* and RT-PCR in *Orobanche cumana*

Fresh tissues of stems and flowers were collected from *P. aegyptiaca*. Total RNA from each sample was extracted with RNAiso Plus (TaKaRa) following the manufacturer’s instructions. RNA concentrations were quantified, and 500 ng of each RNA sample was reverse-transcribed using oligo_(dT) _18_ and RevertAid^TM^ H Minus Reverse Transcriptase (Fermentas) in a total volume of 10 μL. The obtained cDNA samples were diluted to 25 μL. Specific primer pairs, P.aeg-bo1F/P.aeg-bo1R (5′-AGAACCAAGTCTGTGATGGAACC-3′/5′-TCATTTGTAACACCCGAGCCTAT-3′) for the *BO1* gene, and bo2F/bo2R (5′-AGTGCCGATACTGATTACTTCCG-3′/5′-GAGGGTTCGTCACAGACTTGGTT-3′) for the *BO2* gene in *P. aegyptiaca* were designed according to their DNA sequences. *Actin* was again selected as the reference gene for normalizing cDNA concentration variations^59^ and the primer pair Actin-F/Actin-R (5′-CGTGAGAAGATGACGCAGATT-3′/5′-GAACAGCCTGGATAGCAACATAC-3′) was designed accordingly. Quantitative real time-PCR (qRT-PCR) was carried out on an CFX ConnectTM Real-Time System (BIO-RAD) using iTaq^TM^ Universal SYBR Green Supermix (BIO-RAD) following the manufacturer’s instructions. The expression levels were calculated using the comparative CT method. Three biological replicates were run for each gene in stems and flowers. Reverse transcription PCR (RT-PCR) was performed to check the expression of the *BO* transposase genes in *O. cumana* with the primer pair Actin-F/Actin-R.

### TSD-TIR structure search and synteny analysis of genes flanking the *BO* transposase genes in Brassicaceae

To detect the TSD-TIR-transposase-TIR-TSD structure, genomic sequences covering 5000 bp in each direction of the *BO* genes in Brassicaceae species or covering the intergenic segments between two tandem *BO* genes were retrieved. Within these regions, TIRs with 8–23 bp flanked by 8 bp TSDs were searched by using an updated version of the program Transpolator^33^, with a single imperfect nucleotide in the first base of TIRs allowed and TIRs composed of only two types of nucleotides not considered so as to avoid simple repeats. For the colinearity analysis, six *A. thaliana* genes flanking the two *BO* genes with transcriptional activity were selected to search their most similar homologs in other Brassicaceae species.

## Additional Information

**Accession codes:** Sequence data for the Orobancheceae PCR amplification genes have been deposited in the GenBank database (http://www.ncbi.nlm.nih.gov/) under accession number KM037755, KM037756, KT892680-698 and KU187277.

**How to cite this article**: Sun, T. *et al*. Two *hAT* transposon genes were transferred from Brassicaceae to broomrapes and are actively expressed in some recipients. *Sci. Rep.* **6**, 30192; doi: 10.1038/srep30192 (2016).

## Supplementary Material

Supplementary Information
