# Supplementary material for: Two hAT transposon genes were transferred from Brassicaceae to broomrapes and are actively expressed in some recipients
Source: Sci Rep. 2016 Jul 25;6:30192. doi: 10.1038/srep30192 (PMC4958966; doi:10.1038/srep30192)
Supplement: Supplementary Information [file srep30192-s1.pdf]

Two *hAT* transposon genes were transferred from Brassicaceae to broomrapes and are actively expressed in some recipients

Ting Sun[1, 2, 3], Susanne S. Renner[4], Yuxing Xu[1, 3], Yan Qin[1, 5], Jianqiang Wu[1*], and Guiling Sun[1, 2, 5*]

[1]Key Laboratory of Economic Plants and Biotechnology, Yunnan Key Laboratory for Wild Plant Resources, Kunming Institute of Botany, Chinese Academy of Sciences, Kunming 650201, China

[2]Institute of Plant Stress Biology, State Key Laboratory of Cotton Biology, Department of Biology, Henan University, Kaifeng 475004, China

[3]University of the Chinese Academy of Sciences, Beijing 100039, China

[4]Systematic Botany and Mycology, University of Munich (LMU), Munich 80638, Germany

[5]State Key Laboratory of Genetic Resources and Evolution, Kunming Institute of Zoology, Chinese Academy of Sciences, Kunming 650223, China

[*] Corresponding authors

Guiling Sun

Tel: +86 0871-65229552   E-mail address: sung@mail.kib.ac.cn

Jianqiang Wu

Tel: +86 0871-65229562   E-mail address: wujianqiang@mail.kib.ac.cn

Supplementary Table S1. Brassicaceae species included in this study with their tribal placement following Al-Shehbaz (2012).

Species	Tribes	Sources	Number of the *BO* genes
Arabidopsis halleri	Camelineae	http://phytozome.jgi.doe.gov/pz/portal.html	3
Arabidopsis lyrata	Camelineae	http://phytozome.jgi.doe.gov/pz/portal.html	4
Arabidopsis thaliana	Camelineae	http://phytozome.jgi.doe.gov/pz/portal.html	2
Arabis alpina	Arabideae	http://www.ncbi.nlm.nih.gov/genome/	5
Brassica napus	Brassiceae	http://www.ncbi.nlm.nih.gov/genome/	9
Brassica oleracea	Brassiceae	http://www.ocri-genomics.org/bolbase/	6
Brassica rapa	Brassiceae	http://phytozome.jgi.doe.gov/pz/portal.html	5
Raphanus raphanistrum	Brassiceae	http://bioinfo.bti.cornell.edu/cgi-bin/radish/misc/download.cgi	4
Camelina sativa	Camelineae	http://www.ncbi.nlm.nih.gov/genome/	11
Capsella grandiflora	Camelineae	http://phytozome.jgi.doe.gov/pz/portal.html	2
Capsella rubella	Camelineae	http://phytozome.jgi.doe.gov/pz/portal.html	2
Leavenworthia alabamica	Cardamineae	http://brassicadb.org/brad/	2
Sisymbrium irio	Sisymbrieae	http://brassicadb.org/brad/	3
Thellungiella halophila	Eutremeae	http://phytozome.jgi.doe.gov/pz/portal.html	4
Thellungiella salsuginea	Eutremeae	http://phytozome.jgi.doe.gov/pz/portal.html	4
Aethionema arabicum	Aethionemeae	http://brassicadb.org/brad/	0
Boechera stricta	Boechereae	http://phytozome.jgi.doe.gov/pz/portal.html	0
Thellungiella parvula	Eutremeae	http://brassicadb.org/brad/	0

Supplementary Table S2. Species of *Phelipanche* and *Orobanche* used in the genomic PCR amplification, Herbarium voucher information, and the PCR primer sequences and the GenBank accession numbers for the *BO1* and *BO2* genes. The permanent vouchers are deposited in the Munich herbarium (international herbarium acronym: M).

Species	Herbarium voucher	Gene	Primer name	Primer pair	GenBank #
P. aegyptiaca (Pers.) Pomel	S. S. Renner & Yongqing Ma 2853	*BO1*	OhAT-AF2<br>OhAT-AR	5'-GGTGGTTGGGGATAATCTTACTTCA-3'<br>5'-CCCGAGCCTATTGGTTTTTGACAG-3'	KM037755
		BO2	OhAT-BF<br>OhAT-BR	5'-CGCAGGCAGAGAAAGAGCAAAAAT-3'<br>5'-CTGCACATCATGGCGTTCACA-3'	KM037756
P. purpurea (Jacq.) Soják	S. S. Renner 2844	*BO1*	OhAT-AF2<br>OhAT-AR	5'-GGTGGTTGGGGATAATCTTACTTCA-3'<br>5'-CCCGAGCCTATTGGTTTTTGACAG-3'	KT892680
		BO2	3S1<br>4A1	5'-CATAATTGGAAGGTTAGGAAGTGGA-3'<br>5'-TTGGCATATCAAAGTCGTAACCAG-3'	KT892681
P. ramosa (L.) Pomel	S. S. Renner 2852	*BO1*	OhAT-AF2<br>OhAT-AR	5'-GGTGGTTGGGGATAATCTTACTTCA-3'<br>5'-CCCGAGCCTATTGGTTTTTGACAG-3'	KT892682
		BO2	3S1<br>4A1	5'-CATAATTGGAAGGTTAGGAAGTGGA-3'<br>5'-TTGGCATATCAAAGTCGTAACCAG-3'	KT892683
O. cumana Wallr.	S. S. Renner & Yongqing Ma 2854	*BO1*	OhAT-AF2<br>OhAT-AR	5'-GGTGGTTGGGGATAATCTTACTTCA-3'<br>5'-CCCGAGCCTATTGGTTTTTGACAG-3'	KT892693
		BO2	B2F<br>B2R<br>C3F<br>C3R	5'-ATTACCAGGAACAGGACCAA-3'<br>5'-CATGAGCTCTTGGTTCAACA-3'<br>5'-TTTCAGGTTAATGGCTGGTG-3'<br>5'-AACATCACCTTAAGTTCCGC-3'	KT892694
O. amethystea Thuill	S. S. Renner 2848	*BO1*	OhAT-AF2<br>OhAT-AR	5'-GGTGGTTGGGGATAATCTTACTTCA-3'<br>5'-CCCGAGCCTATTGGTTTTTGACAG-3'	KT892684
		BO2	6S1<br>3A1	5'-ATAGCATCGAAATAATCGCGTCC-3'<br>5'-CTTCATCAGATGGTTGGTCCTGT-3'	KT892685
O. crenata Forssk.	S. S. Renner 2849	*BO1*	OhAT-AF2<br>OhAT-AR	5'-GGTGGTTGGGGATAATCTTACTTCA-3'<br>5'-CCCGAGCCTATTGGTTTTTGACAG-3'	KT892686
		BO2	C3F<br>C3R	5'-TTTCAGGTTAATGGCTGGTG-3'<br>5'-AACATCACCTTAAGTTCCGC-3'	KU187277
O. salvia F.W.Schultz	S. S. Renner 2850	*BO1*	OhAT-AF2<br>OhAT-AR	5'-GGTGGTTGGGGATAATCTTACTTCA-3'<br>5'-CCCGAGCCTATTGGTTTTTGACAG-3'	KT892687
		BO2	6S1<br>3A1	5'-ATAGCATCGAAATAATCGCGTCC-3'<br>5'-CTTCATCAGATGGTTGGTCCTGT-3'	KT892688
O. hederae Duby	S. S. Renner 2842	*BO1*	OhAT-AF2<br>OhAT-AR	5'-GGTGGTTGGGGATAATCTTACTTCA-3'<br>5'-CCCGAGCCTATTGGTTTTTGACAG-3'	KT892689
		BO2	OhAT-BF<br>OhAT-BR	5'-CGCAGGCAGAGAAAGAGCAAAAAT-3'<br>5'-CTGCACATCATGGCGTTCACA-3'	KT892690
O. lucorum A.Braun ex F.W.Schultz	S. S. Renner 2843	*BO1*	OhAT-AF2<br>OhAT-AR	5'-GGTGGTTGGGGATAATCTTACTTCA-3'<br>5'-CCCGAGCCTATTGGTTTTTGACAG-3'	KT892691
		BO2	6S1<br>3A1	5'-ATAGCATCGAAATAATCGCGTCC-3'<br>5'-CTTCATCAGATGGTTGGTCCTGT-3'	KT892692
O. gracilis Sm.	S. S. Renner 2851	*BO1*	OhAT-AF2<br>OhAT-AR	5'-GGTGGTTGGGGATAATCTTACTTCA-3'<br>5'-CCCGAGCCTATTGGTTTTTGACAG-3'	KT892695
		BO2	6S1<br>3A1	5'-ATAGCATCGAAATAATCGCGTCC-3'<br>5'-CTTCATCAGATGGTTGGTCCTGT-3'	KT892696

```
Orohed1_Orobanche_hederae       ------------------------------------------------------------------------------------------------------------------------------------------------------
Orosal1_Orobanche_salviae       ------------------------------------------------------------------------------------------------------------------------------------------------------
Oroame1_Orobanche_amethystea    ------------------------------------------------------------------------------------------------------------------------------------------------------
Orocre1_Orobanche_crenata       ------------------------------------------------------------------------------------------------------------------------------------------------------
Phepur1_Phelipanche_purpurea    ------------------------------------------------------------------------------------------------------------------------------------------------------
Oroluc1_Orobanche_lucorum       ------------------------------------------------------------------------------------------------------------------------------------------------------
Pheram1_Phelipanche_ramosa      ------------------------------------------------------------------------------------------------------------------------------------------------------
Orocum1_Orobanche_cumana        ------------------------------------------------------------------------------------------------------------------------------------------------------
Pheaeg1_Phelipanche_aegyptiaca  ------------------------------------------------------------------------------------------------------------------------------------------------------
Orogra1_Orobanche_gracilis      ------------------------------------------------------------------------------------------------------------------------------------------------------
Oroaus1_Orobanche_austrohispan  ------------------------------------------------------------------------------------------------------------------------------------------------------
Pheram2_Phelipanche_ramosa      ------------------------------------------------------------------------------------------------------------------------------------------------------
Phepur2_Phelipanche_purpurea    ------------------------------------------------------------------------------------------------------------------------------------------------------
Pheaeg2_Phelipanche_aegyptiaca  ------------------------------------------------------------------------------------------------------------------------------------------------------
Orocre2_Orobanche_crenata       ------------------------------------------------------------------------------------------------------------------------------------------------------
Oroaus2_Orobanche_austrohispan  ------------------------------------------------------------------------------------------------------------------------------------------------------
Orohed2_Orobanche_hederae       ------------------------------------------------------------------------------------------------------------------------------------------------------
Orocum2_Orobanche_cumana        ------------------------------------------------------------------------------------------------------------------------------------------------------
Orogra2_Orobanche_gracilis      AATTATATAATATATGTAAATTACAGTAAAAAGTTAATCAATTTAACTGATCAAAAAAAAAATGGACATATAAATAGAAACGGAAGGAGTATAAACATACTCATGTAATAGCATCGAAATAATCGCGTCCGTCCACTGTTTGCTTGACCG
Oroame2_Orobanche_amethystea    -----------------------------------------------------------------------------------------------------------ATAGCATCGAAATAATCGCGTCCGTCCACTGTTTGCCTGACCG
Oroluc2_Orobanche_lucorum       -----------------------------------------------------------------ATAGCATCGAAATAATCGCGTCCCATTCCGGAGGACCAAAATAAAAAATAAAAATAATCGCGTCCGTCCACTGTTTGCTTGACCG
Orosal2_Orobanche_salviae       -----------------------------------------------------------------ATAGCATCGAAATAATCGCGTCCCAT-CCGGAGGACCAAAATAAAAAATAAAAATAATCGCGTCCGTCCACTGTTTGCTTGACCG

Orohed1_Orobanche_hederae       ------------------------------------------------------------------------------------------------------------------------------------------------------
Orosal1_Orobanche_salviae       ------------------------------------------------------------------------------------------------------------------------------------------------------
Oroame1_Orobanche_amethystea    ------------------------------------------------------------------------------------------------------------------------------------------------------
Orocre1_Orobanche_crenata       ------------------------------------------------------------------------------------------------------------------------------------------------------
Phepur1_Phelipanche_purpurea    ------------------------------------------------------------------------------------------------------------------------------------------------------
Oroluc1_Orobanche_lucorum       ------------------------------------------------------------------------------------------------------------------------------------------------------
Pheram1_Phelipanche_ramosa      ------------------------------------------------------------------------------------------------------------------------------------------------------
Orocum1_Orobanche_cumana        ------------------------------------------------------------------------------------------------------------------------------------------------------
Pheaeg1_Phelipanche_aegyptiaca  ------------------------------------------------------------------------------------------------------------------------------------------------------
Orogra1_Orobanche_gracilis      ------------------------------------------------------------------------------------------------------------------------------------------------------
Oroaus1_Orobanche_austrohispan  ------------------------------------------------------------------------------------------------------------------------------------------------------
Pheram2_Phelipanche_ramosa      ------------------------------------------------------------------------------------------------------------------------------------------------------
Phepur2_Phelipanche_purpurea    ------------------------------------------------------------------------------------------------------------------------------------------------------
Pheaeg2_Phelipanche_aegyptiaca  ------------------------------------------------------------------------------------------------------------------------------------------------------
Orocre2_Orobanche_crenata       ------------------------------------------------------------------------------------------------------------------------------------------------------
Oroaus2_Orobanche_austrohispan  -----------------------------------------------------------------------------------ATGCATAATGCGATCTTGATACCGATGCATCATCTTCTTTTTTTA----------------------
Orohed2_Orobanche_hederae       ------------------------------------------------------------------------------------------------------------------------------------------------------
Orocum2_Orobanche_cumana        ------------------------------------------------------------------------------------------------------------------------------------------------------
Orogra2_Orobanche_gracilis      AAACGTCCAATCCAACGCATCTGATAATCACCAAACTCAAAGAAAGCAAAAAAGAAGCCAACCATGAAAAGCCACGTCCGTGTGTAGCTCCAGTAGCATTCCCGCCTTAAACCCATCTCTTCTTCCTCAATTGCAAACCCCAACAGTTTC
Oroame2_Orobanche_amethystea    ATACGTCCAATCCAACGCCTCTGATAATCACCAAACTCAA-G---------------------------------------------------TAGTATCCCCGCCTTAAA-CCATCTCTTCTTCCCCAATCGC-AACCCTAACAGTTTC
Oroluc2_Orobanche_lucorum       AAACGTCCAATCCAACGCCTCTGATAATCACCAAACTCAAAGAAAGCAAAAAAGAAGCCAACCATGAAAAGCCACGTCCGTGTGTAGCTCCAATAGCATTCCCGCCTTAAA-CCATCTCTTCTTCCTCAATCGCAAACCCTAACAGTTTC
Orosal2_Orobanche_salviae       AAACGTCCAATCCAACGCCTCTGATAATCACCAAACTCAAAGAAAGCAAAAAAGAAGCCAACCATGAAAAGCCACGTCCGTGTGTAGCTCCAATAGCATTCCCGCCTTAAA-CCATCTCTTCTTCCTCAATCGCAAACCCTAACAGTTTC

Orohed1_Orobanche_hederae       ------------------------------------------------------------------------------------------------------------------------------------------------------
Orosal1_Orobanche_salviae       ------------------------------------------------------------------------------------------------------------------------------------------------------
Oroame1_Orobanche_amethystea    ------------------------------------------------------------------------------------------------------------------------------------------------------
Orocre1_Orobanche_crenata       ------------------------------------------------------------------------------------------------------------------------------------------------------
Phepur1_Phelipanche_purpurea    ------------------------------------------------------------------------------------------------------------------------------------------------------
Oroluc1_Orobanche_lucorum       ------------------------------------------------------------------------------------------------------------------------------------------------------
Pheram1_Phelipanche_ramosa      ------------------------------------------------------------------------------------------------------------------------------------------------------
Orocum1_Orobanche_cumana        ------------------------------------------------------------------------------------------------------------------------------------------------------
Pheaeg1_Phelipanche_aegyptiaca  -----------------------------------------------------------------------ATGAGGAAGAAACTGAAGTTGG-ACGACACTGAGGAGGTGGTTGGGGATAATCTTACTTCACTACAGGAAAAGGAGAAA
Orogra1_Orobanche_gracilis      -----------------------------------------------------------------------ATGAGGAAGAAACTAAAGTTGG-ACGACTCTGAGGAGGTGGTTGGGGATAATCTTACTTCACTAGAGGAAAAGGAGAAA
Oroaus1_Orobanche_austrohispan  -----------------------------------------------------------------------ATGAGGAAGAAACTAAAGTTGG-ACGACTCTGAGGAGGTGGTTGGGGATAATCTTACTTCACTAGAGGAAAAGGAGAAA
Pheram2_Phelipanche_ramosa      ------------------------------------------------------------------------------------------------------------------------------------------------------
Phepur2_Phelipanche_purpurea    ------------------------------------------------------------------------------------------------------------------------------------------------------
Pheaeg2_Phelipanche_aegyptiaca  --------------------------------------ATGGAAGACGGTGATCTCGCCTCCGGTTCAGATGTGAAAGAGAAACAAAAATCGG-ACAATGAGCAGATTGTGGTGGATAGTCGAATCCCTACGCAGGCAGAGAAAGAGCAA
Orocre2_Orobanche_crenata       ------------------------------------------------------------------------------------------------------------------------------------------------------
Oroaus2_Orobanche_austrohispan  --------------------------------------------------------------------GATGTGAAAGAGAAACAAAAATCGG-ACAATGAGCAGATTGTGGTGGATAGTCGAATCCCTACGCAGGCAGAGAAAGAGCAA
Orohed2_Orobanche_hederae       ------------------------------------------------------------------------------------------------------------------------------------------------------
Orocum2_Orobanche_cumana        ------------------------------------------------------------------------------------------------------------------------------------------------------
Orogra2_Orobanche_gracilis      TCTCTAGTTCCTCTCCTCCGCAAAACAGCAACGTTTCTATGGAAGACGGTGATCTCGCCTCCGGTTCAGATGTGAAAGAGAAACAAAAATCGG-ACAATGAGCAGATTGTGGTGGATAGTCGAATCCCTACGCAGGCAGAGAAAGAGCAA
Oroame2_Orobanche_amethystea    TCTCTAGTTCCTCTCCCCCGCAAAACAGCAACATTTCTATGGAGGACGGTGATCTCGCCTCCGGTTCAGATGTGAAAGAGAAACAAAAATCGG-ACAATGAGCAGATTGTGGTGGATAGTCGAATCCCTACGCAGGCAGAGAAAGAGCAA
Oroluc2_Orobanche_lucorum       TCTCTAGTTCCTCTCCCCCGCAAAACAGCAACGTTTCTATGGAAGACGGTGATCTCGCCTCCGGTTCAGATGTGAAAGAGAAACAAAAATCGGGACAATGATCAGATTGTGGTGGATAGTCGAATCCCTATGCAGGCAGAGAAAGAGCAA
Orosal2_Orobanche_salviae       TCTCTAGTTCCTCTCCCCCGCAAAACAGCAACGTTTCTATGGAAGACGGTGATCTCGCCTCCGGTTCAGATGTGAAAGAGAAACAAAAATCGG-ACAATGATCAGATTGTGGTGGATAGTCGAATCCCTATGCAGGCAGAGAAAGAGCAA
```

```
Orohed1_Orobanche_hederae      AGAGGTCATAAAGCTTTGGCCAATATGTTCATTCATGGTGGGATTAATCCTAATACCATTAAAAGCCATTCCTTTGCAAAGTTTATGCGGCTTCTCAACCCGAAT------TTTCCTCTT-AGTGTGTCTCTAGTCGAGAAGGAAGTTTT
Orosal1_Orobanche_salviae      AGAGGTCATAAAGCTTTGGCCAATTTGTTCATTCATGGTGGGATTAATCCTAATACCATTAAAATCCATTCCTTTGCAAAGTTTATGCAGCTTCTCAACCCGAAT------TCTCGTCTT-AGTGTGTCTCTAGTCGAGAAGGAAGTTTT
Oroame1_Orobanche_amethystea   ------------AGCTTTGGCCATATGTTCATTCATGGTGGGATTAATCCTAATACCATTAAAAGCCATTCCTTTGCAAAGTTTATGCGGCTTCTCAACCCGAAT------TTTCCTCTT-AGTGTGTCTCTAGTCGAGAAGGAAGTTTT
Orocre1_Orobanche_crenata      ------------AAGCTTGGCCATATGTTCATTCATGGTGGGATTAATCCTAATACCATTAAAAGCCATTCATTTGCAAAGTTTATGCGGCTTCTCAACCCGAAT------TTTCCTCTT-AGTGTGTCTCTAGTCGAGAAGGAAGTTTT
Phepur1_Phelipanche_purpurea   --------TAAAGCTTTGGCCAAAATGTTCATTCATGGTGGGATTAATCCTAATACCATTAAAAGCCATTCCTTTGCAAAGTGTATGCGGCTTCTCAACCCGAACCCGATTTTTCGTCTT-AGTGTGTCTCTAGTCGAGAAGGAAGTTTT
Oroluc1_Orobanche_lucorum      -----------------------TTTGTTCATTCATGGTGGGATTAATCCTAATACCATTAAAATCCATTCCTTTGCAAAGTTTATGCAGCTTCTCAACCCGAAT------TCTCGTCTT-AGTGTGTCTCTAGTCGAGAAGGAAGTTTT
Pheram1_Phelipanche_ramosa     ---------AAAGCTTTGGCCAAATCGTTCATTCATGGTGGGATTAATCCTAATACCATTAAAAGCCATTCCTTTGCAAAGCTTATGCGGCTTCTCAACCCGAAT------TTTTGTCTT-ATTGTGTCTCTAGTCGAGAAGGGAGTTTT
Orocum1_Orobanche_cumana       ------------GCTTTGGCCAATTCGTTCATTCATGGTGGGATTAATCCTAATACCATTAAAAGCCCTTCCTTTGCAAAGTTTATGCGGCTTCTCAACCCGGAT------TTTTGTCTT-AGTGTGTCTCTAGTCGAGAAGGAAGTTTT
Pheaeg1_Phelipanche_aegyptiaca GAAGGTCATAAAGCTTTGGCCAAATCGTTCATTCATGGTGGGATTAATCCTAATACCATTAAAAGCCATTCCTTTGCAAAGCTTATGCGGCTTCTCAACCCGAAT------TTTTGTCTT-AGTGTGTCTCTAGTCGAGAAGGAAGTTTT
Orogra1_Orobanche_gracilis     GAAGGTCATAAAGCTTTGGCCAATTTGTTCATTCATGGTGGGATTAATCCTAATACCATTAAAAGCCATTCCTTTGCAAAGTTTATGCGGATTCTCAACCCGAAT------TCTCGTCTT-AGTGTGTCTCTAGTCGAGAAGGAAGTTTT
Oroaus1_Orobanche_austrohispan GAAGGTCATAAAGCTTTGGCCAATTTGTTCATTCATGGTGGGATTAATCCTAATACCATTAAAAGCCATTCCTTTGCAAAGTTTATGCGGCTTCTCAACCCGAAT------TCTCGTCTT-AGTGTGTCTCTAGTCGAGAAGGAAGTTTT
Pheram2_Phelipanche_ramosa     ------------------------------------------------------------------------------------------------------------------------------------------------------
Phepur2_Phelipanche_purpurea   ------------------------------------------------------------------------------------------------------------------------------------------------------
Pheaeg2_Phelipanche_aegyptiaca AAATATAAGAGATCTATCGCCGGATGGCTTATTCGTACTGCTCGTGTGCCTCCTCAT---------------------TCCTTTATGCAGCGTATGGGCCCGCTC------TTGCCTTTT-GCTGTTTCTCAACTCGAGAGAGAAGTTCT
Orocre2_Orobanche_crenata      ------------------------------------------------------------------------------------------------------------------------------------------------------
Oroaus2_Orobanche_austrohispan AAATGTAAGAGATCTATCGCCGGATGGCTCATTCGTACTGCTCGTGTGCCTCCTCAT---------------------TCCTTTATGCAGCGTATGGGCCCGCTC------TTGCCTTTC-GGTGGTTCTCAACTCGAGAGAGAAGTTCT
Orohed2_Orobanche_hederae      -------------------------------TTCGTACTGCTCGTGTGCCTCCTCAT---------------------TCCTTTATGCAGCGTATGGGCCCGCTC------TTGCCTTTC-GGTGTTTCTCAACTCGAGAGAGAAGTTCT
Orocum2_Orobanche_cumana       ------------------------------------------------------------------------------------------------------------------------------------------------------
Orogra2_Orobanche_gracilis     AAATGTAAGAGATCTATCGCCGGATGGCTCATTCGTACTGCTCGTGTGCCTCCTCAT---------------------TCCTTTATGCAGCGTATGGGCCCGCTC------TTGCCTTTC-GGTGGTTCTCAACTCGAGAGAGAAGTTCT
Oroame2_Orobanche_amethystea   AAATGTAAGAGATCTATTGCCGGATGGCTCATTCGTACTGCTCGTGTGCCTCGTCAT---------------------TCCTTTATGCAGCGTATGGGCCCACTC------TTGCCTTTC-GGTGTTTCTCAACTCGAGAGAGAAGTTCT
Oroluc2_Orobanche_lucorum      AAATGTAGGAGATCTATTGCCGGATGGCTCATTCGTACTACTCGTGTGCCTCCTCAT---------------------TCCTTTATGCAGCGTATAGGCCCGCTC------TTGCCTTTTCGGTGTTTCTCAACTCGAAAGAGAAGTTCT
Orosal2_Orobanche_salviae      AAATGTAAGAGATCTATTGCCGGATGGCTCATTCGTACTACTCGTGTGCCTCCTCAT---------------------TCCTTTATGCAGCGTATAGGCCCGCTC------TTGCCTTTC-GGTGGTTCTCAACTCGAAAGAGAAGTTCT

Orohed1_Orobanche_hederae      GGAGATACACGAAGAATGTAAAGAGAAAGCTAAGAAATTTCTCAAGGATTTCCAAGGGAAGGTAACACTCTCTTAC---------------------GAATGGATGGTTCTTGGGCATGACTGGACCAGGGATTATATTAAAGGTCCTAT
Orosal1_Orobanche_salviae      GGAGATACACGAAGAATTTAAAGAGAAAGCTAAGAAATTTCTCAAGGATTTCCAAGGGAAGGTAACACTCTCTTAC---------------------GAATGGATGGTTCTTGGGCATGATTGGACCAGGGATTATATTAAATGTCCTAT
Oroame1_Orobanche_amethystea   GGAGATACACGAAGAATGTAAAGAGAAAGCTAAGAAATTTCTCAAGGATTTCCAAGGGAAGGTAACACTCTCTTAC---------------------GAATGGATGGTTCTTGGGCATGACTGGACCAGGGATTATATTAAAGGTCCTAT
Orocre1_Orobanche_crenata      GGAGATACACGAAGAATGTAAAGCGAAAGCTAAGAAATTTCTCAAGGATTTCCAAGGGAAGGTAACACTCTCTTAC---------------------GAATGGATGGTTCTTGGGCATGACTGGACCAGGGATTATATTAAAGGTCCTAT
Phepur1_Phelipanche_purpurea   GGAGATACACGAAGAATGTAAAGAGAAAGCTAAGAAATTTCTCAAGGATTTCCAAGGGAAGGTAACACTCTCTTAC---------------------GAATGGATGGTTCTTGGGCATGACTGGACCAGGGATTATATTAAAGGTCCTAT
Oroluc1_Orobanche_lucorum      GGAGATACACGAAGAATTTAAAGAGAAAGCTAAGAAATTTCTCAAGGATTTCCAAGGGAAGGTAACACTCTCTTAC---------------------GAATGGATGGTTCTTGGGCATGATTGGACCAGGGATTATATTAAATGTCCTAT
Pheram1_Phelipanche_ramosa     GGAGATACACGAAGAATGTAAAGAGAAAGCTAAGAAATTTCTCAAGGATTTCCAAGGGAAGGTAACACTCTCTTAC---------------------GAATGGATGGTTCTTGGGCATGACTGGACCAGGGATCATATTGAAGGTCCTAT
Orocum1_Orobanche_cumana       GGAGATACACGAAGAATGTAAAGAGAAAGCTAAGAAATTTCTCAAGGATTTCCAAGGGAAGGTAACACTCTCTTAC---------------------GAATGGATGGTTCTTGGGCATGACTGGACCAGGGATTATATTAAAGGTCCTAT
Pheaeg1_Phelipanche_aegyptiaca GGAGATACACGAAGAATGTAAAGAGAAAGCTAAGAAATTTCTCAAGGATTTCCAAGGGAAGGTAACACTCTCTTAC---------------------GAATGGATGGTTCTTGGGCATGACTGGACCAGGGATCATATTGAAGGTCCTAT
Orogra1_Orobanche_gracilis     GGAGATACACGAAGAATGTAAAGAGAAAGCTAAGAAATTTCTCAAGGATTTCCAAGGAAAGGTAACACTCTCTTAC---------------------GAATGGATGGTTCTTGGGCATGACTGGACCAGGGATTATATTAAAAGTCCTAT
Oroaus1_Orobanche_austrohispan GGAGATACACGAAGAATGTAAAGAGAAAGCTAAGAAATTTCTCAAGGATTTCCAAGGAAAGGTAACACTCTCTTAC---------------------GAATGGATGGTTCTTGGGCATGACTGGACCAGGGATTATATTAAAAGTCCTAT
Pheram2_Phelipanche_ramosa     ------------------------------------------------------------------------------------------------------------------------------------------------------
Phepur2_Phelipanche_purpurea   ------------------------------------------------------------------------------------------------------------------------------------------------------
Pheaeg2_Phelipanche_aegyptiaca GAAGATACATCAAGAACGTAAAGACAAGGCCAAGAAATTTCTCAAGTGTTTTGATGGGAAATTAACAATCTCTTATGACCGTGTGGCTTATGATGATGAATGGGAGGCCTATGACGAAGAATGGGACTGTTCATCCAGAGATGGCCCTGT
Orocre2_Orobanche_crenata      ------------------------------------------------------------------------------------------------------------------------------------------------------
Oroaus2_Orobanche_austrohispan GAAGATACATCAAGAACGTAAAGACAAGGTCAAGAAATTTCT----------GATGGGAAATTAACAATCTCTTATGACCGTGTGGCTTATGATGATGAATGGGAGGCCTATGACGAAGAATGGGACTGTTCCTCCAGAGATGGCCCTGT
Orohed2_Orobanche_hederae      GAAGATACATCAAGAACGTAA-GACAAGGCCAAGAAATTTCTCAAGTGTTTTGATGGGAAATTAACAATCTCTTATGACCATGTGGCTTATGATGATGAATGGGAGGCCTATGACGAATAATGGGACTGTTCATCCAGAGATG-------
Orocum2_Orobanche_cumana       ------------------------------------------------------------------------------------------------------------------------------------------------------
Orogra2_Orobanche_gracilis     GAAGATACATCAAGAACGTAAAGACAAGGTCAAGAAATTTCT----------GATGGGAAATTAACAATCTCTTATGACCGTGTGGCTTATGATGATGAATGGGAGGCCTATGACGAAGAATGGGACTGTTCATCCAGAGATGGCCCTGT
Oroame2_Orobanche_amethystea   GAAGATACATCAAGAACGTAAAGACAAGGCCAAGAAGTTTCTCAAGTGTTTTGATGGGAAATTAACAATCTCTTATGACCGTGTGGGTTATGATGATGAATGGGAGGCCTATGACGCAGAATGGGACTGTTCATCCAGAGATGGCCCTGT
Oroluc2_Orobanche_lucorum      GAAGATACATCAAGAACGTAAAGACAAGGCCAAGAAATTTCTCAAGTGTTTTGATGGGAAATTAACAATCTCTTATGACCGTGTGGCTTATGATGATGAATGTGAGGCTTATGATGAAGAATGGGACTGTTCATCCAGAGATGGCCCTGT
Orosal2_Orobanche_salviae      GAAGATACATCAAGAACGTAAAGACAAGGCCAAGAAATTTCTCAAGTGTTTTGATGGGAAATTAACAATCTCTTATGACCGTGTGGCTTATGATGATGAATGTGAGGCTTATGATGAAGAATGGGACTGTTCATCCAGAGATGGCCCTGT

Orohed1_Orobanche_hederae      CTTGCACGAGGATTTCGTCTGTATCACTGCTCATTTCATTGATGATAACTGGAAGGTCAGGAAATGGATCCTCGGGTACACTACTGACGAGTCTGTATCTCTGG----------------------------------------------
Orosal1_Orobanche_salviae      CTTGCACGAGGATTTCGTCTGTATCACTGCTCATTTCATTGATGATAACTGGAAGGTCAGGAAATGGATCCTCGGGTACACTACTGACGAGTCTGTATCTCTGG----------------------------------------------
Oroame1_Orobanche_amethystea   CTTGCACGAGGATTTCGTCTGTATCACTGCTCATTTCATTGATGATAACTGGAAGGTCAGGAAATGGATCCTCGGGTACACTACTGACGAGTCTGTATCTCTGG----------------------------------------------
Orocre1_Orobanche_crenata      CTTGCACGAGGATTTCGTCTGTATCACTGCTCATTTCATTGATGATAACTGGAAGGTCAGGAAATGGATCCTCGGGTACACTACTGACGAGTCTGTATCTCTGG----------------------------------------------
Phepur1_Phelipanche_purpurea   CTTGCACGAGGATTTCGTCTGTATCACTGCTCATTTCATTGATGATAACTGGAAGGCCAGGAAATGGATCCTCGGGTACACTACTGACGAGTCTGTATCTCTGG----------------------------------------------
Oroluc1_Orobanche_lucorum      CTTGCACGAGGATTTCGTCTGTATCACTGCTCATTTCATTGATGATAACTGGAAGGTCAGGAAATGGATCCTCGGGTACACTACTGACGAGTCTGTATCTCTGG----------------------------------------------
Pheram1_Phelipanche_ramosa     CTTGCACGAGGATTTCATTTGTATCACTGCTCATTTCATTGATGATAACTGGAAGGTCAGGAAATGGATCCTCGGGTACACTACTGACGAGTCTGTATCTCCGG----------------------------------------------
Orocum1_Orobanche_cumana       CTTGCACGAGGATTTCGTCTGTATCACTGCTCATTTCATTGATGATAACTGGAAGGTCAGGAAATGGATCCTCGGGTACACTACTGACGAGTCTGTACCCCTGG----------------------------------------------
Pheaeg1_Phelipanche_aegyptiaca CTTGCACGAGGATTTCATCTGTATCACTGCTCATTTCATTGATGATAACTGGAAGGTCAGGAAATGGATCCTCGGGTACACTACTGACGAGTCTGTATCTCCGG----------------------------------------------
Orogra1_Orobanche_gracilis     CTTGCATGAGGATTTCGTCTGTATCACTGCTCATTTCATTGATGATAACTGGAAGGTCAGGAAACGGATCCTCGGGTACACTACTGACGAGTCTGTATCTCTGG----------------------------------------------
Oroaus1_Orobanche_austrohispan CTTGCATGAGGATTTCGTCTGTATCACTGCTCATTTCATTGATGATAACTGGAAGGTCAGGAAACGGATCCTCGGGTACACTACTGACGAGTCTGTATCTCTGG----------------------------------------------
Pheram2_Phelipanche_ramosa     ------------------------------------------------------------------------------------------------------------------------------------------------------
Phepur2_Phelipanche_purpurea   --------------------------------------------------------TTAGGAAGTGGATCTTGGGGTACTCTCCTATCGAGTTTGTACGAGAGG----------------------------------------------
Pheaeg2_Phelipanche_aegyptiaca TTTGCACAAGAGTTTCGTCTGTATAAGTGTCCATTTCGTTGATCATAATTGGAAGGTTAGGAAGTGGATCTTGGGGTACTCTCCTATCGAGTTTGTACGAGAGG----------------------------------------------
Orocre2_Orobanche_crenata      ------------------------------------------------------------------------------------------------------------------------------------------------------
Oroaus2_Orobanche_austrohispan TTTGCACAAGAATT--GTCTGTATAAGTGTCCATTTCGTTGATCATAATTGGAAGGTTAGGAAGTGGATCTTGGGGTACTCTCCTATCGAGTTTGTACGAGAGG----------------------------------------------
Orohed2_Orobanche_hederae      -----------------------------TCCATTTCGTTTATCATAATTGGAAGGTTAGGAAGTAGATCTTGGGGTACTCACCTATCGAGTTTGTGCGAGAGGAAAAACTTGAGGTTTACAAATTTAGGAACGTTATTTGGATTATGAG
Orocum2_Orobanche_cumana       ------------------------------------------------------------------------------------------------------------------------------------------------------
Orogra2_Orobanche_gracilis     TTTGCACAAGAGTTTCGTCTGTATAAGTGTCCATTTCGTTGATCATAATTGGAAGGTTAGGAAGTGGATCTTGGGGTACTCTCCTATCGAGTTTGTACGAGAGG----------------------------------------------
Oroame2_Orobanche_amethystea   TTTGCACAAGAGTTTCGTCTGTATAAGTGTCCATTTCGTTGATCATAATTGGAAGGTTAGGAAGTGGATCTTGGGGTACTCTCGTATCGAGTTTGTACGAAAGG----------------------------------------------
Oroluc2_Orobanche_lucorum      TTTGCACAAGAGTTTCGTCTGTATAAGTGTCCATTTCGTTGATCACAATTTTAAGGTTAGGAAGTGGATTTTGGGGTACTCTCCTATCGGGTTTGTACGAGAGG----------------------------------------------
Orosal2_Orobanche_salviae      TTTGCACAAGAGTTTCGTCTGTATAAGTGTCCATTTCGTTGATCACAATTTTAAGGTTAGGAAGTGGATTTTGGGGTACTCTCCTATCGAGTTTGTACGAGAGG----------------------------------------------
```

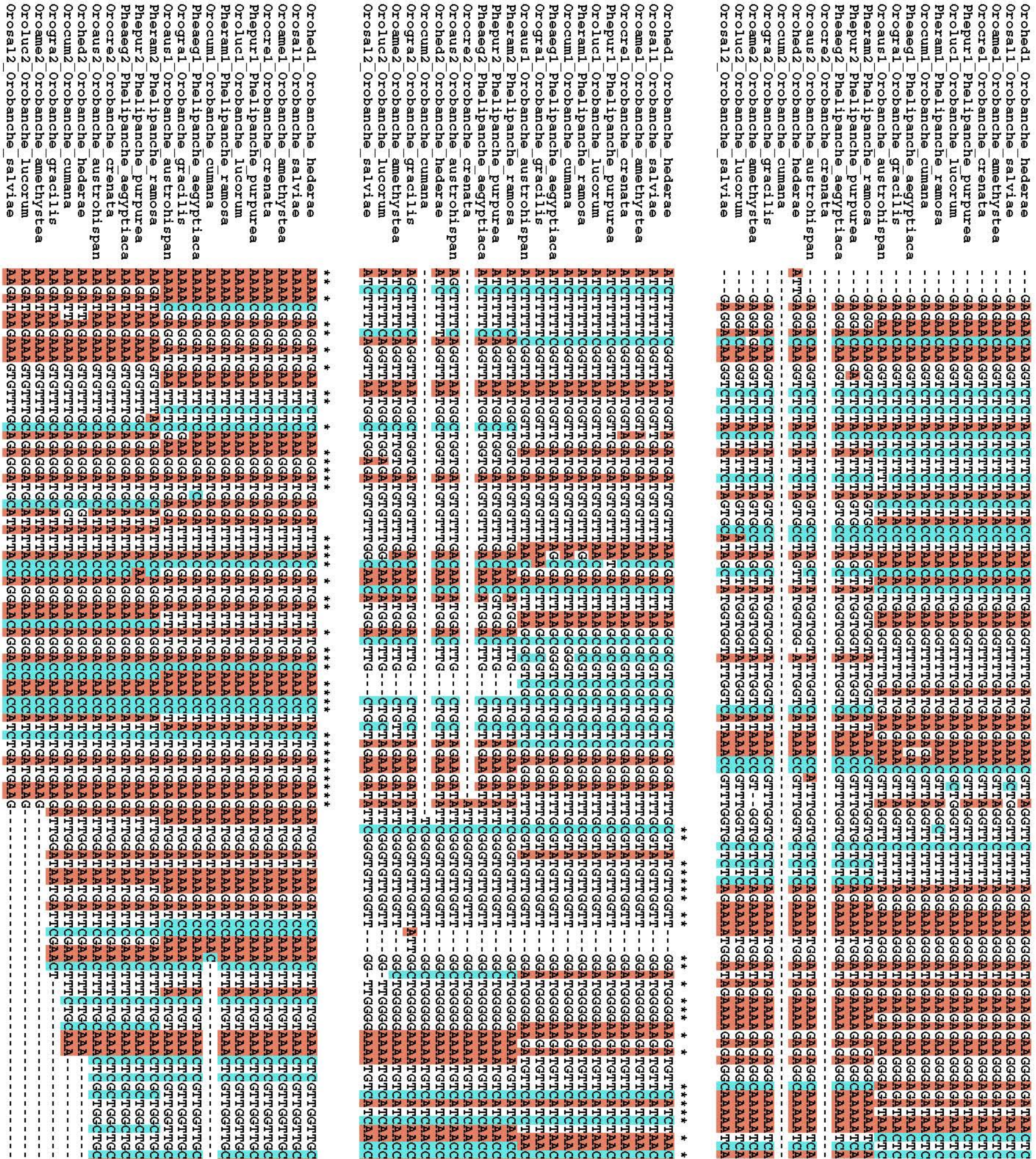
Orohed1_Orobanche_hederae
Orosal1_Orobanche_salviae
Oroame1_Orobanche_amethystea
Orocre1_Orobanche_crenata
Phepur1_Phelipanche_purpurea
Oroluc1_Orobanche_lucorum
Pheram1_Phelipanche_ramosa
Orocum1_Orobanche_cumana
Pheaeg1_Phelipanche_aegyptiaca
Orogra1_Orobanche_gracilis
Oroaus1_Orobanche_austrohispan
Pheram2_Phelipanche_ramosa
Phepur2_Phelipanche_purpurea
Pheaeg2_Phelipanche_aegyptiaca
Orocre2_Orobanche_crenata
Oroaus2_Orobanche_austrohispan
Orohed2_Orobanche_hederae
Orocum2_Orobanche_cumana
Orogra2_Orobanche_gracilis
Oroame2_Orobanche_amethystea
Oroluc2_Orobanche_lucorum
Orosal2_Orobanche_salviae

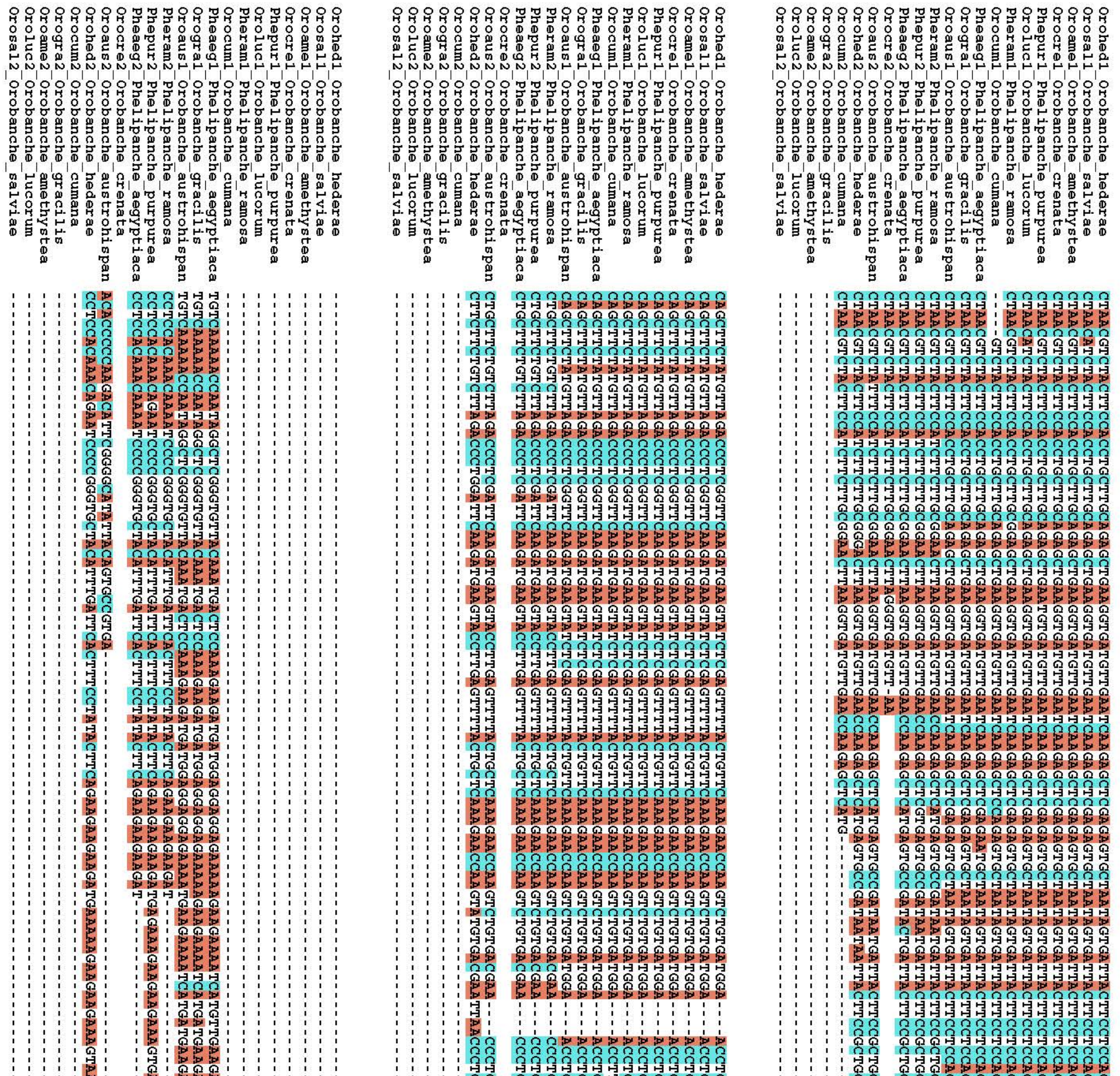
Orohed1_Orobanche_hederae
Orosal1_Orobanche_salviae
Oroame1_Orobanche_amethystea
Orocre1_Orobanche_crenata
Phepur1_Phelipanche_purpurea
Oroluc1_Orobanche_lucorum
Pheram1_Phelipanche_ramosa
Orocum1_Orobanche_cumana
Pheaeg1_Phelipanche_aegyptiaca
Orogra1_Orobanche_gracilis
Oroaus1_Orobanche_austrohispan
Pheram2_Phelipanche_ramosa
Phepur2_Phelipanche_purpurea
Pheaeg2_Phelipanche_aegyptiaca
Orocre2_Orobanche_crenata
Oroaus2_Orobanche_austrohispan
Orohed2_Orobanche_hederae
Orocum2_Orobanche_cumana
Orogra2_Orobanche_gracilis
Oroame2_Orobanche_amethystea
Oroluc2_Orobanche_lucorum
Orosal2_Orobanche_salviae
Orohed1_Orobanche_hederae
Orosal1_Orobanche_salviae
Oroame1_Orobanche_amethystea
Orocre1_Orobanche_crenata
Phepur1_Phelipanche_purpurea
Oroluc1_Orobanche_lucorum
Pheram1_Phelipanche_ramosa
Orocum1_Orobanche_cumana
Pheaeg1_Phelipanche_aegyptiaca
Orogra1_Orobanche_gracilis
Oroaus1_Orobanche_austrohispan
Pheram2_Phelipanche_ramosa
Phepur2_Phelipanche_purpurea
Pheaeg2_Phelipanche_aegyptiaca
Orocre2_Orobanche_crenata
Oroaus2_Orobanche_austrohispan
Orohed2_Orobanche_hederae
Orocum2_Orobanche_cumana
Orogra2_Orobanche_gracilis
Oroame2_Orobanche_amethystea
Oroluc2_Orobanche_lucorum
Orosal2_Orobanche_salviae
Orohed1_Orobanche_hederae
Orosal1_Orobanche_salviae
Oroame1_Orobanche_amethystea
Orocre1_Orobanche_crenata
Phepur1_Phelipanche_purpurea
Oroluc1_Orobanche_lucorum
Pheram1_Phelipanche_ramosa
Orocum1_Orobanche_cumana
Pheaeg1_Phelipanche_aegyptiaca
Orogra1_Orobanche_gracilis
Oroaus1_Orobanche_austrohispan
Pheram2_Phelipanche_ramosa
Phepur2_Phelipanche_purpurea
Pheaeg2_Phelipanche_aegyptiaca
Orocre2_Orobanche_crenata
Oroaus2_Orobanche_austrohispan
Orohed2_Orobanche_hederae
Orocum2_Orobanche_cumana
Orogra2_Orobanche_gracilis
Oroame2_Orobanche_amethystea
Oroluc2_Orobanche_lucorum
Orosal2_Orobanche_salviae

```
Orohed1_Orobanche_hederae     ------------------------------------------------------------------------------------------------------------------------------------------------------
Orosal1_Orobanche_salviae     ------------------------------------------------------------------------------------------------------------------------------------------------------
Oroame1_Orobanche_amethystea  ------------------------------------------------------------------------------------------------------------------------------------------------------
Orocre1_Orobanche_crenata     ------------------------------------------------------------------------------------------------------------------------------------------------------
Phepur1_Phelipanche_purpurea  ------------------------------------------------------------------------------------------------------------------------------------------------------
Oroluc1_Orobanche_lucorum     ------------------------------------------------------------------------------------------------------------------------------------------------------
Pheram1_Phelipanche_ramosa    ------------------------------------------------------------------------------------------------------------------------------------------------------
Orocum1_Orobanche_cumana      ------------------------------------------------------------------------------------------------------------------------------------------------------
Pheaeg1_Phelipanche_aegyptiaca ------------------------------------------------------------------------------------------------------------------------------------------------------
Orogra1_Orobanche_gracilis    ACAAGAAGAAGGAGAAGACGGTTATGATGATGAAGAAGAAGAAGATTATGACGAAGAACATGAAGAAGGAGAAGAAGATTATGATTCTGATTCATCAGCAGCAGAAGAAAGGGCAGCACAAAAGGATAGAGAAAAGAAGCCCGACGCTTG
Oroaus1_Orobanche_austrohispan ACAAGAAGAAGGAGAAGACGGTTATGATGATGAAGAAGAAGAAGATTATGACGAAGAACATGAAGAAGGAGAAGAAGATTATGATTCTGATTCATCAGCAGCAGAAGAAAGGGCAGCACAAAAGGATAGAGAAAAGAAGCCCGACGCTTG
Pheram2_Phelipanche_ramosa    AGGTGATAAAGAATGTGTAGAGGAAGAAGAAGGTGGAGAAGAT---------------------------------------------------------------------------AAAGACAAGGAAGAGAAGAAGCCTGATGCATA
Phepur2_Phelipanche_purpurea  AGGTGATAAAGAATGTGTAGAGGAAGAAGAAGGTGGAGAAGATGT-------------------------------------------------------------------------AAAGACAAGGAAGAGAAGAAGCCTGATGCATA
Pheaeg2_Phelipanche_aegyptiaca AGGTGATAAAGAATGTGTAGAGGAAGAAGAAGGTGGAGAAGAT---------------------------------------------------------------------------AAAGACAAGGAAGAGAAGAAGCCTGATGCATA
Orocre2_Orobanche_crenata
Oroaus2_Orobanche_austrohispan ------------------------------------------------------------------------------------------------------------------------------------------------------
Orohed2_Orobanche_hederae     AGGTGATAAAGAATGTGTAGAGGAAGAAGAAGGTGGAGAAGACCATA------------------------------------------------------------------------AAGACAAGGAAGAGAAGAAACCTGATGCATA
Orocum2_Orobanche_cumana      ------------------------------------------------------------------------------------------------------------------------------------------------------
Orogra2_Orobanche_gracilis    ------------------------------------------------------------------------------------------------------------------------------------------------------
Oroame2_Orobanche_amethystea  ------------------------------------------------------------------------------------------------------------------------------------------------------
Oroluc2_Orobanche_lucorum     ------------------------------------------------------------------------------------------------------------------------------------------------------
Orosal2_Orobanche_salviae     ------------------------------------------------------------------------------------------------------------------------------------------------------

Orohed1_Orobanche_hederae     ------------------------------------------------------------------------------------------------------------------------------------------------------
Orosal1_Orobanche_salviae     ------------------------------------------------------------------------------------------------------------------------------------------------------
Oroame1_Orobanche_amethystea  ------------------------------------------------------------------------------------------------------------------------------------------------------
Orocre1_Orobanche_crenata     ------------------------------------------------------------------------------------------------------------------------------------------------------
Phepur1_Phelipanche_purpurea  ------------------------------------------------------------------------------------------------------------------------------------------------------
Oroluc1_Orobanche_lucorum     ------------------------------------------------------------------------------------------------------------------------------------------------------
Pheram1_Phelipanche_ramosa    ------------------------------------------------------------------------------------------------------------------------------------------------------
Orocum1_Orobanche_cumana      ------------------------------------------------------------------------------------------------------------------------------------------------------
Pheaeg1_Phelipanche_aegyptiaca ------------------------------------------------------------------------------------------------------------------------------------------------------
Orogra1_Orobanche_gracilis    CAAGGATTTTGCTTTCTTTCAAGAGTTTCTCAAATTTGAAGGATCTCCAAGAGAGTTTGGTGAATCAGAGCTTGATTCCTACCTCAAAGAACCGGTTATGGAGTGGAACAAAGACTTTAAAGCGTTGGACTGGTGGAGAGAAGAGGGTCA
Oroaus1_Orobanche_austrohispan CAAGGATTTTGCTTTCTTTCAAGAGTTTCTCAAATTTGAAGGATCTCCAAGAGAGTTTGGTGAATCAGAGCTTGATTCCTACCTCAAAGAACCGGTTATGGAGTGGAACAAAGACTTTAAAGCGTTGGACTGGTGGAGAGAAGAGGGTCA
Pheram2_Phelipanche_ramosa    TGAAGATTTTGTTTTGTTTCAAGAATATCTCAAGTTTGAAGAATCTTCAAGAGAGTTTGATGAATCAGAAGTTGATTCCTATCTCAAAGAGCCAGTTTTGGAGTGGGACAAGGACTTTAAAGCATTGGAATGGTGGAGAGAAGAGAGCCA
Phepur2_Phelipanche_purpurea  TGAAGATTTTGTTTTGTTTCAAGAATATCTCAAGTTTGAAGAATCTTCAAGAGAGTTTGATGAATCAGAAGTTGATTCCTATCTCAAAGAGCCAGTTTTGGAGTGGGACAAAGACTTTAAAGCATTGGAATGGTGGAGAGAAGAGAGCCA
Pheaeg2_Phelipanche_aegyptiaca TGAAGATTTTGTTTTGTTTCAAGAATATCTCAAGTTTGAAGAATCTTCAAGAGAGTTTGATGAATCAGAAGTTGATTCCTATCTCAAAGAGCCAGTTTTGGAGTGGGACAAGGACTTTAAAGCATTGGAATGGTGGAGAGAAGAGAGCCA
Orocre2_Orobanche_crenata
Oroaus2_Orobanche_austrohispan ------------------------------------------------------------------------------------------------------------------------------------------------------
Orohed2_Orobanche_hederae     TGAAGATTTTGTTTTGTTTCAAGAATATCTCAAGTTTGAAGAATCTTCAAGAGAGTTTGATGAATCAGAACTTGATTCCTATCTCAAAGAACCAGTTTTGGAGTGGGAC------TTTAAAGCATTGGAATGGTGGATAGAAGAGAGCCA
Orocum2_Orobanche_cumana      ------------------------------------------------------------------------------------------------------------------------------------------------------
Orogra2_Orobanche_gracilis    ------------------------------------------------------------------------------------------------------------------------------------------------------
Oroame2_Orobanche_amethystea  ------------------------------------------------------------------------------------------------------------------------------------------------------
Oroluc2_Orobanche_lucorum     ------------------------------------------------------------------------------------------------------------------------------------------------------
Orosal2_Orobanche_salviae     ------------------------------------------------------------------------------------------------------------------------------------------------------

Orohed1_Orobanche_hederae     ------------------------------------------------------------------------------------------------------------------------------------------------------
Orosal1_Orobanche_salviae     ------------------------------------------------------------------------------------------------------------------------------------------------------
Oroame1_Orobanche_amethystea  ------------------------------------------------------------------------------------------------------------------------------------------------------
Orocre1_Orobanche_crenata     ------------------------------------------------------------------------------------------------------------------------------------------------------
Phepur1_Phelipanche_purpurea  ------------------------------------------------------------------------------------------------------------------------------------------------------
Oroluc1_Orobanche_lucorum     ------------------------------------------------------------------------------------------------------------------------------------------------------
Pheram1_Phelipanche_ramosa    ------------------------------------------------------------------------------------------------------------------------------------------------------
Orocum1_Orobanche_cumana      ------------------------------------------------------------------------------------------------------------------------------------------------------
Pheaeg1_Phelipanche_aegyptiaca ------------------------------------------------------------------------------------------------------------------------------------------------------
Orogra1_Orobanche_gracilis    CAAGTATCCAATCCTCTCTCGAGTAGCTCGTGACATTCTCTCAATTCCAATCTCACGAGCCACGTCCTACAATGCTTATGTTTCGTACAGAAGAGAACCTCCTGAGTTTGTTCTCTCCTTGGAACCCAAAGTTGCCAACGCCATGATGTG
Oroaus1_Orobanche_austrohispan CAAGTATCCAATCCTCTCTCGAGTAGCTCGTGACATTCTCTCAATTCCAATCTCACGAGCCACGTCCTACAATGCTTATGTTTCGTACAGAAGAGAACCTCCTGAGTTTGTTCTCTCCTTGGAACCCAAAGTTGCCAACGCCATGATGTG
Pheram2_Phelipanche_ramosa    AAAGTATCCAATCCTCTCCCGAATAGCTCGTGATATTCTCTCGATTCCAATCTCGCGCGCCACATCGTATGTCGCATATGTTGCTGACAGAAGAGAGTGTCCTGAATTTGTTGTCACCATGAAAGCCAAACTG-----------------
Phepur2_Phelipanche_purpurea  AAAGTATCCAATCCTCTCCCGAATAGCTCGTGACATACTCTCCATTCCAATCTCACGCGCCACATCGTATGTCGCATATGTTGCTGACAAAAGAGTGTGTCCTGAATTT-----------------------------------------
Pheaeg2_Phelipanche_aegyptiaca AAAGTATCCAATCCTCTCCCGAATAGCTCGTGACATTCTCTCCATTCCAATCTCGCGCGCCACATCGTATGTCGCATATGTTGCTGACAAAAGAGAGTGTCCTGAATTTGTTGTCACCATGAAAGCCAAACTTGTGAACGCCATGATGTG
Orocre2_Orobanche_crenata
Oroaus2_Orobanche_austrohispan ------------------------------------------------------------------------------------------------------------------------------------------------------
Orohed2_Orobanche_hederae     AAAGTATCCAATCCTCTCCCGAATAGCTCGTGACATTCTCTCCATTCCAATCTCGCGCGCCACATCGTATGTCGCATATGTTGCTGACAAAAGAGAGTGTTCT-----------------------------------------------
Orocum2_Orobanche_cumana      ------------------------------------------------------------------------------------------------------------------------------------------------------
Orogra2_Orobanche_gracilis    ------------------------------------------------------------------------------------------------------------------------------------------------------
Oroame2_Orobanche_amethystea  ------------------------------------------------------------------------------------------------------------------------------------------------------
Oroluc2_Orobanche_lucorum     ------------------------------------------------------------------------------------------------------------------------------------------------------
Orosal2_Orobanche_salviae     ------------------------------------------------------------------------------------------------------------------------------------------------------
```

```
Orohed1_Orobanche_hederae       -------------------------
Orosal1_Orobanche_salviae       -------------------------
Oroame1_Orobanche_amethystea    -------------------------
Orocre1_Orobanche_crenata       -------------------------
Phepur1_Phelipanche_purpurea    -------------------------
Oroluc1_Orobanche_lucorum       -------------------------
Pheram1_Phelipanche_ramosa      -------------------------
Orocum1_Orobanche_cumana        -------------------------
Pheaeg1_Phelipanche_aegyptiaca  -------------------------
Orogra1_Orobanche_gracilis      CAGCCAAAAATGGTTACAACTTTGA
Oroaus1_Orobanche_austrohispan  CAGCCAAAAATGGTTACAACTTTGA
Pheram2_Phelipanche_ramosa      -------------------------
Phepur2_Phelipanche_purpurea    -------------------------
Pheaeg2_Phelipanche_aegyptiaca  CAGCAAGCGCTGGTCACGACTTTGA
Orocre2_Orobanche_crenata       -------------------------
Oroaus2_Orobanche_austrohispan  -------------------------
Orohed2_Orobanche_hederae       -------------------------
Orocum2_Orobanche_cumana        -------------------------
Orogra2_Orobanche_gracilis      -------------------------
Oroame2_Orobanche_amethystea    -------------------------
Oroluc2_Orobanche_lucorum       -------------------------
Orosal2_Orobanche_salviae       -------------------------
```

Supplementary Figure S1. The alignment of the *BO* transposase genes from *Orobanche* and *Phelipanche* obtained by searching the draft genomes and genomic PCR. The species names are indicated after the sequence IDs. Dashes indicated the sequences were incomplete or gaps introduced in the alignment. Background colors indicated the degree of conservation of the sites.

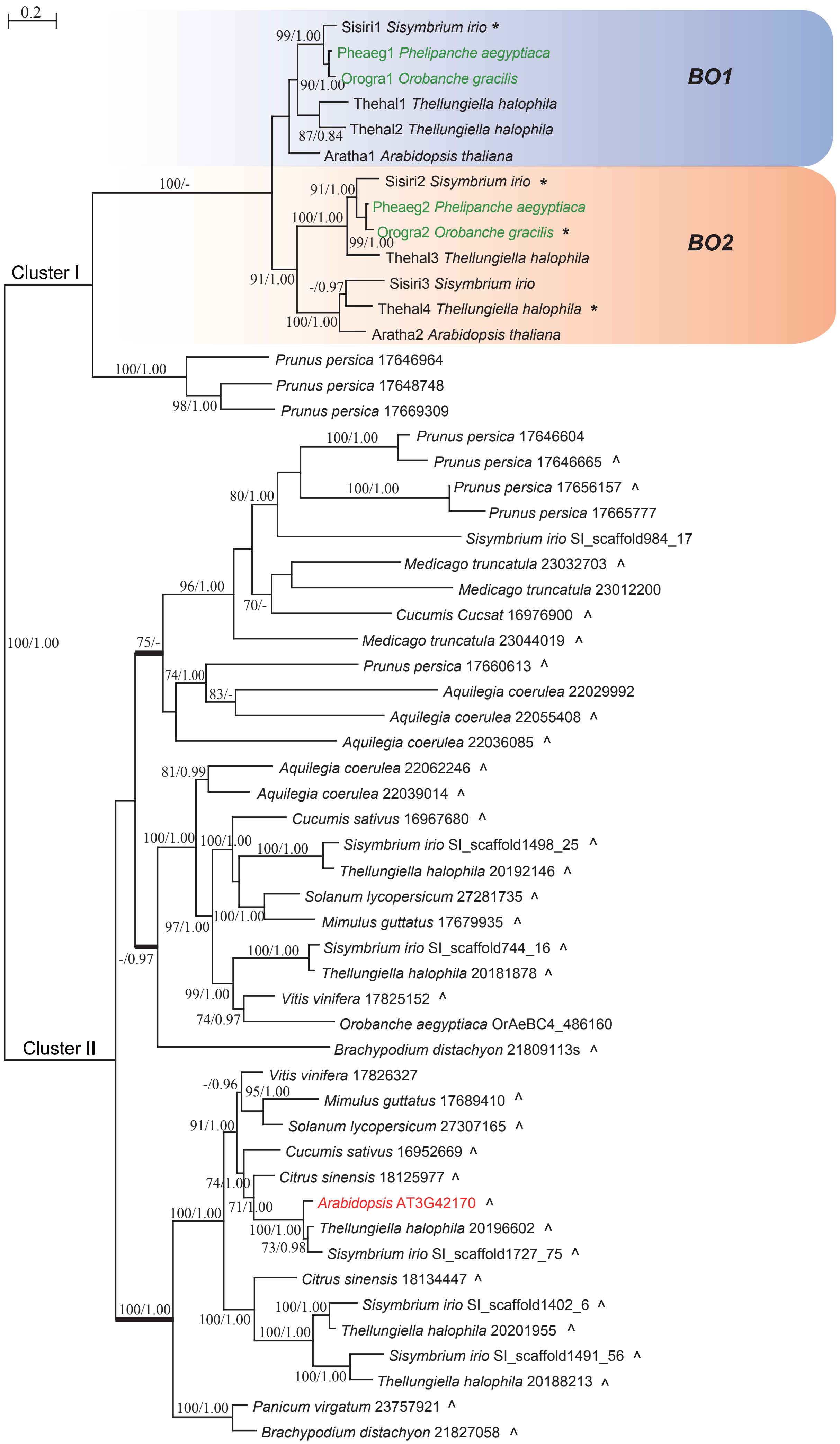

0.2
BO1
BO2
Cluster I
Cluster II
Sisiri1 Sisymbrium irio *
Pheaeg1 Phelipanche aegyptiaca
Orogra1 Orobanche gracilis
Thehal1 Thellungiella halophila
Thehal2 Thellungiella halophila
Aratha1 Arabidopsis thaliana
Sisiri2 Sisymbrium irio *
Pheaeg2 Phelipanche aegyptiaca
Orogra2 Orobanche gracilis *
Thehal3 Thellungiella halophila
Sisiri3 Sisymbrium irio
Thehal4 Thellungiella halophila *
Aratha2 Arabidopsis thaliana
Prunus persica 17646964
Prunus persica 17648748
Prunus persica 17669309
Prunus persica 17646604
Prunus persica 17646665 ^
Prunus persica 17656157 ^
Prunus persica 17665777
Sisymbrium irio SI_scaffold984_17
Medicago truncatula 23032703 ^
Medicago truncatula 23012200
Cucumis Cucsat 16976900 ^
Medicago truncatula 23044019 ^
Prunus persica 17660613 ^
Aquilegia coerulea 22029992
Aquilegia coerulea 22055408 ^
Aquilegia coerulea 22036085 ^
Aquilegia coerulea 22062246 ^
Aquilegia coerulea 22039014 ^
Cucumis sativus 16967680 ^
Sisymbrium irio SI_scaffold1498_25 ^
Thellungiella halophila 20192146 ^
Solanum lycopersicum 27281735 ^
Mimulus guttatus 17679935 ^
Sisymbrium irio SI_scaffold744_16 ^
Thellungiella halophila 20181878 ^
Vitis vinifera 17825152 ^
Orobanche aegyptiaca OrAeBC4_486160
Brachypodium distachyon 21809113s ^
Vitis vinifera 17826327
Mimulus guttatus 17689410 ^
Solanum lycopersicum 27307165 ^
Cucumis sativus 16952669 ^
Citrus sinensis 18125977 ^
Arabidopsis AT3G42170 ^
Thellungiella halophila 20196602 ^
Sisymbrium irio SI_scaffold1727_75 ^
Citrus sinensis 18134447 ^
Sisymbrium irio SI_scaffold1402_6 ^
Thellungiella halophila 20201955 ^
Sisymbrium irio SI_scaffold1491_56 ^
Thellungiella halophila 20188213 ^
Panicum virgatum 23757921 ^
Brachypodium distachyon 21827058 ^

Supplementary Figure S2. The phylogeny of the BO family of *hAT* transposon and related protein in flowering plants. Clusters I and II are marked above the branches. Numbers above branches show bootstrap support values for maximum likelihood and posterior probability values for MrBayes analysis. Dashes indicate values lower than 70% in the maximum likelihood analysis and 0.90 in the MrBayes analysis. The *BO1* and *BO2* genes from representative species of Brassicaceae and Orobanchaceae are highlighted with blue and orange background, and those from Orobanchaceae are in green. Asterisks in the nodes indicate putative pseudogenes. The three well-supported branches in Cluster II are highlighted with increased thickness. The *DAYSLEEPER* gene of *A. thaliana* is in red. Carets indicate the sequences containing an additional C2H2 type BED-zinc finger domain in the N terminus. The Phytozome IDs or GenBank Gi numbers of genes are placed following the species names.

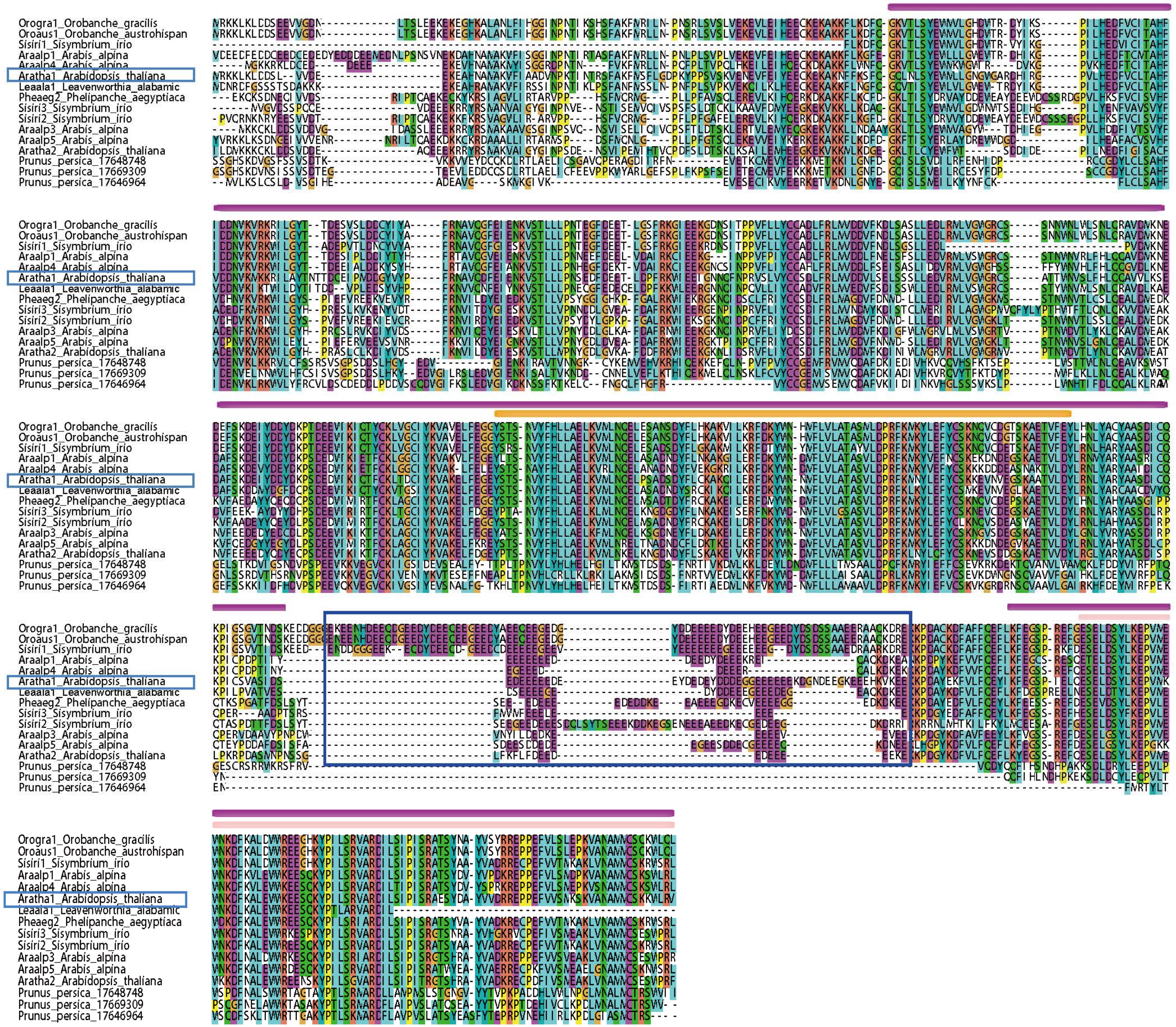

Supplementary Figure S3. The domain structures of the *BO* transposase genes in Brassicaceae, *Orobanche*, and *Phelipanche*, and the related homologs in peach.

The predicted amino acid sequences were used for the alignment using ClustalX2. The bars in different color above the alignment indicate the three domains, the *hAT*-like transposase domain is yellow, the *hAT* dimerization domain is pink, and the ribonuclease H-like domain is purple. The insertion within the ribonuclease H-like domain with varied lengths, which is uniquely present in the *BO* genes in Brassicaceae and *Orobanche* and is absent in the genes of peach, is in a blue frame.

A

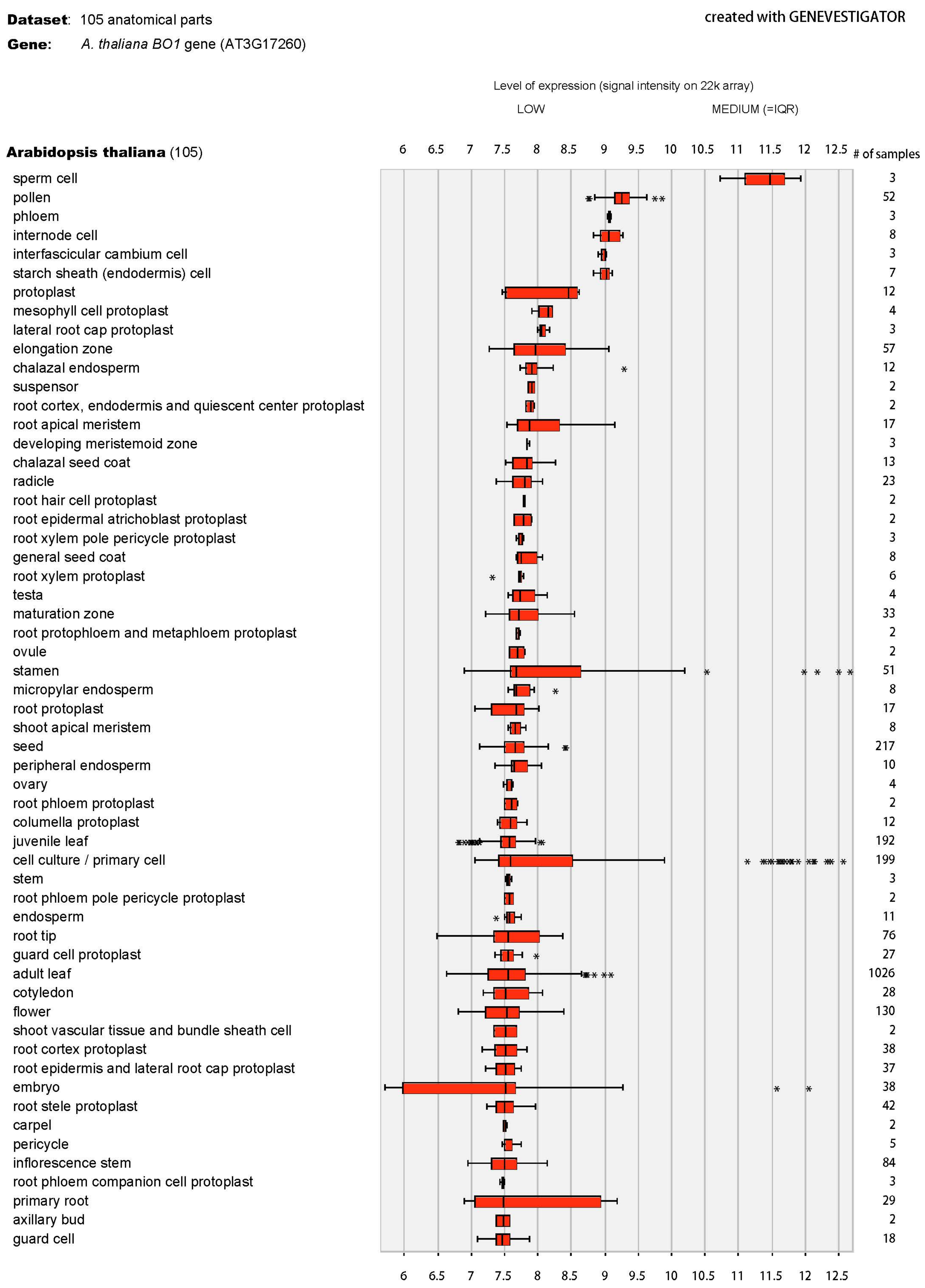
Dataset: 105 anatomical parts
Gene: A. thaliana BO1 gene (AT3G17260)
created with GENEVESTIGATOR
Level of expression (signal intensity on 22k array)
LOW
MEDIUM (=IQR)
Arabidopsis thaliana (105)
6 6.5 7 7.5 8 8.5 9 9.5 10 10.5 11 11.5 12 12.5
# of samples
sperm cell 3
pollen 52
phloem 3
internode cell 8
interfascicular cambium cell 3
starch sheath (endodermis) cell 7
protoplast 12
mesophyll cell protoplast 4
lateral root cap protoplast 3
elongation zone 57
chalazal endosperm 12
suspensor 2
root cortex, endodermis and quiescent center protoplast 2
root apical meristem 17
developing meristemoid zone 3
chalazal seed coat 13
radicle 23
root hair cell protoplast 2
root epidermal atrichoblast protoplast 2
root xylem pole pericycle protoplast 3
general seed coat 8
root xylem protoplast 6
testa 4
maturation zone 33
root protophloem and metaphloem protoplast 2
ovule 2
stamen 51
micropylar endosperm 8
root protoplast 17
shoot apical meristem 8
seed 217
peripheral endosperm 10
ovary 4
root phloem protoplast 2
columella protoplast 12
juvenile leaf 192
cell culture / primary cell 199
stem 3
root phloem pole pericycle protoplast 2
endosperm 11
root tip 76
guard cell protoplast 27
adult leaf 1026
cotyledon 28
flower 130
shoot vascular tissue and bundle sheath cell 2
root cortex protoplast 38
root epidermis and lateral root cap protoplast 37
embryo 38
root stele protoplast 42
carpel 2
pericycle 5
inflorescence stem 84
root phloem companion cell protoplast 3
primary root 29
axillary bud 2
guard cell 18
6 6.5 7 7.5 8 8.5 9 9.5 10 10.5 11 11.5 12 12.5

Dataset: 105 anatomical parts

created with GENEVESTIGATOR

Gene: *A. thaliana BO1* gene (AT3G17260)

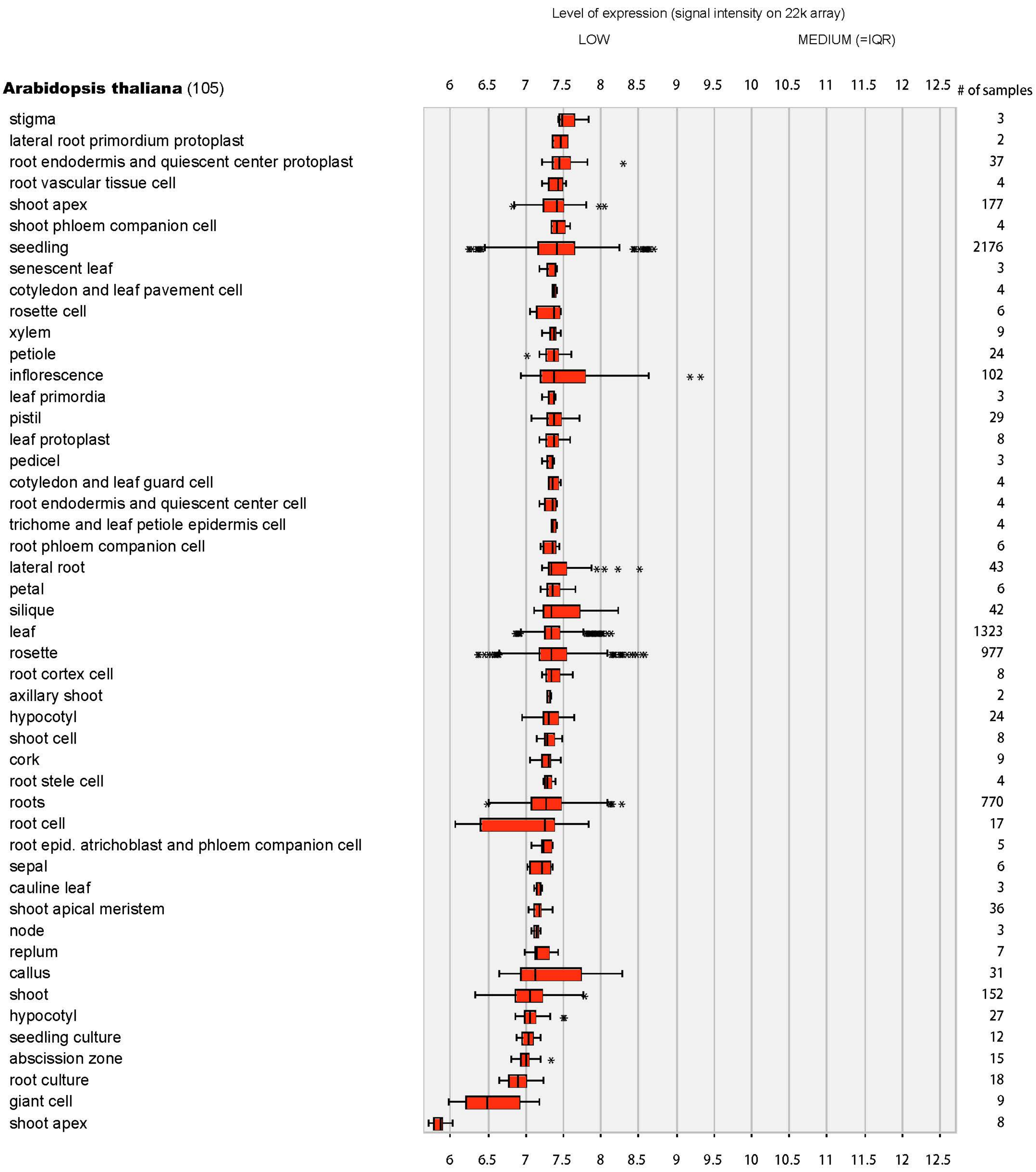

B

Dataset: 105 anatomical parts

Gene: *A. thaliana BO2* gene (AT3G17290)

created with GENEVESTIGATOR

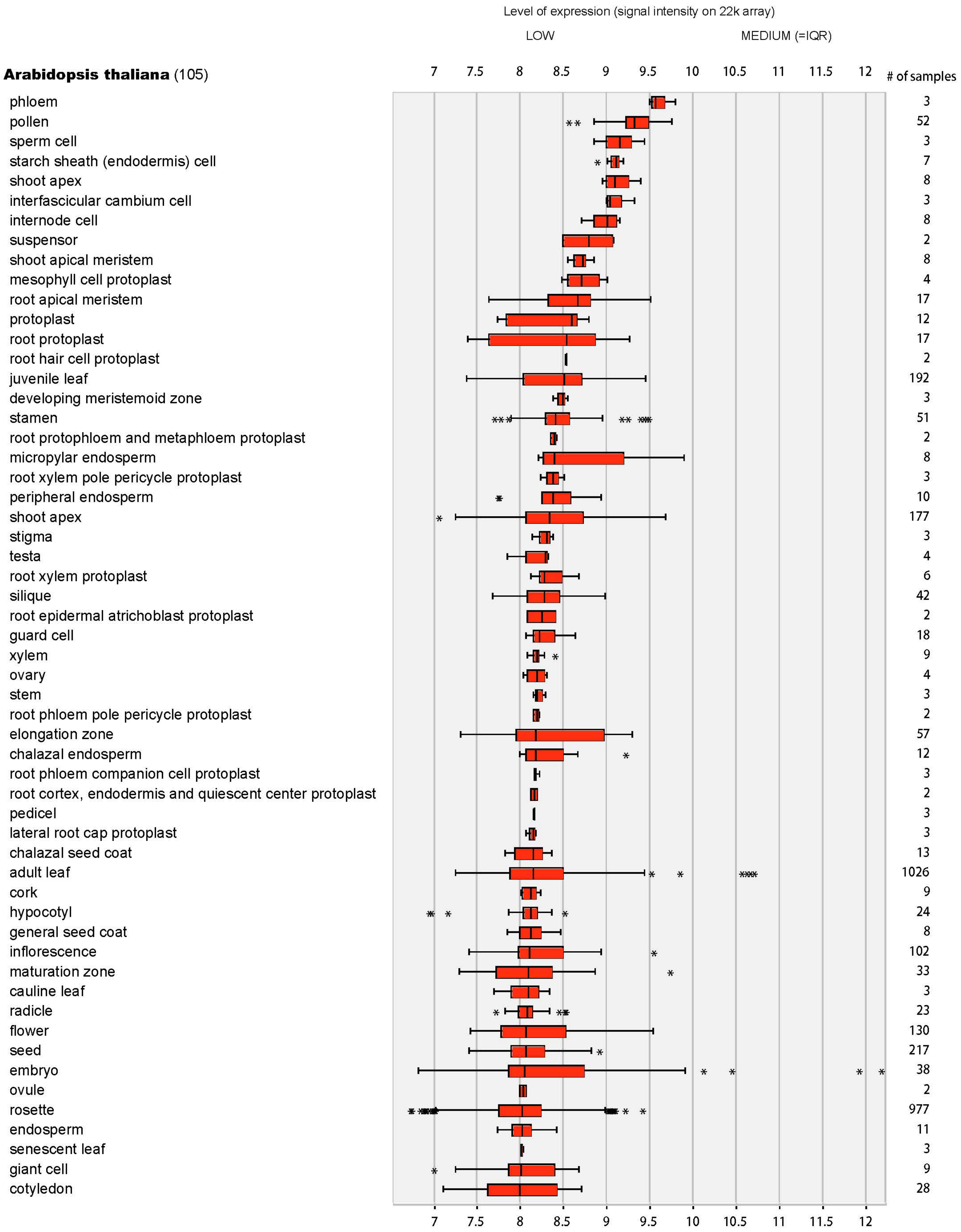

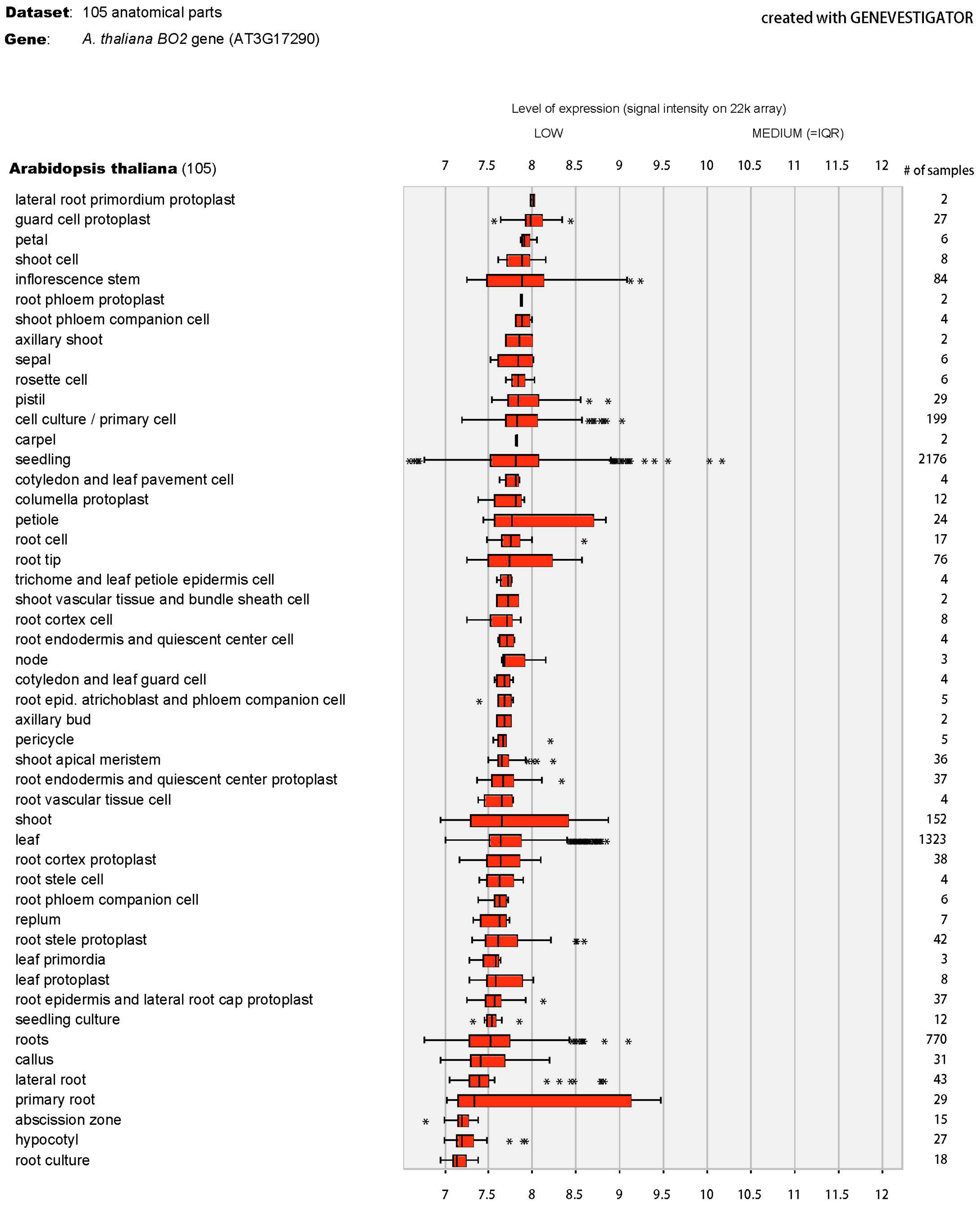

Supplementary Figure S4. The expression levels of *A. thaliana BO1* and *BO2* genes in different tissues.

Web-based expression analyses of *A. thaliana BO1* gene (AT3G17260, A) and *BO2* gene (AT3G17290, B) in in different tissues were performed using GENEVESTIGATOR v3 (https://www.genevestigator.com/gv/).
